# NF-κB mediated regulation of tumor cell proliferation in hypoxic microenvironment

**DOI:** 10.3389/fphar.2023.1108915

**Published:** 2023-02-20

**Authors:** Shubham Rastogi, Sara Aldosary, Abdulaziz S. Saeedan, Mohd. Nazam Ansari, Manjari Singh, Gaurav Kaithwas

**Affiliations:** ^1^ Department of Pharmaceutical Sciences, School of Biosciences and Biotechnology, Babasaheb Bhimrao Ambedkar University, Lucknow, Uttar Pradesh, India; ^2^ Department of Pharmaceutical Sciences, King Faisal University, Al-Ahsa, Saudi Arabia; ^3^ Department of Pharmacology, College of Pharmacy, Prince Sattam Bin Abdulaziz University, Al-Kharj, Saudi Arabia; ^4^ Department of Pharmaceutical Sciences, Assam Central University, Silchar, India

**Keywords:** lactate shuttle, cancer, HIF-1α, NF-κB, hypoxia, PHD2

## Abstract

Hypoxia is caused by a cancer-promoting milieu characterized by persistent inflammation. NF-κB and HIF-1α are critical participants in this transition. Tumor development and maintenance are aided by NF-κB, while cellular proliferation and adaptability to angiogenic signals are aided by HIF-1α. Prolyl hydroxylase-2 (PHD-2) has been hypothesized to be the key oxygen-dependent regulator of HIF-1α and NF-transcriptional B’s activity. Without low oxygen levels, HIF-1α is degraded by the proteasome in a process dependent on oxygen and 2-oxoglutarate. As opposed to the normal NF-κB activation route, where NF-κB is deactivated by PHD-2-mediated hydroxylation of IKK, this method actually activates NF-κB. HIF-1α is protected from degradation by proteasomes in hypoxic cells, where it then activates transcription factors involved in cellular metastasis and angiogenesis. The Pasteur phenomenon causes lactate to build up inside the hypoxic cells. As part of a process known as lactate shuttle, MCT-1 and MCT-4 cells help deliver lactate from the blood to neighboring, non-hypoxic tumour cells. Non-hypoxic tumour cells use lactate, which is converted to pyruvate, as fuel for oxidative phosphorylation. OXOPHOS cancer cells are characterized by a metabolic switch from glucose-facilitated oxidative phosphorylation to lactate-facilitated oxidative phosphorylation. Although PHD-2 was found in OXOPHOS cells. There is no clear explanation for the presence of NF-kappa B activity. The accumulation of the competitive inhibitor of 2-oxo-glutarate, pyruvate, in non-hypoxic tumour cells is well established. So, we conclude that PHD-2 is inactive in non-hypoxic tumour cells due to pyruvate-mediated competitive suppression of 2-oxo-glutarate. This results in canonical activation of NF-κB. In non-hypoxic tumour cells, 2-oxoglutarate serves as a limiting factor, rendering PHD-2 inactive. However, FIH prevents HIF-1α from engaging in its transcriptional actions. Using the existing scientific literature, we conclude in this study that NF-κB is the major regulator of tumour cell growth and proliferation *via* pyruvate-mediated competitive inhibition of PHD-2.

## Graphical Abstract

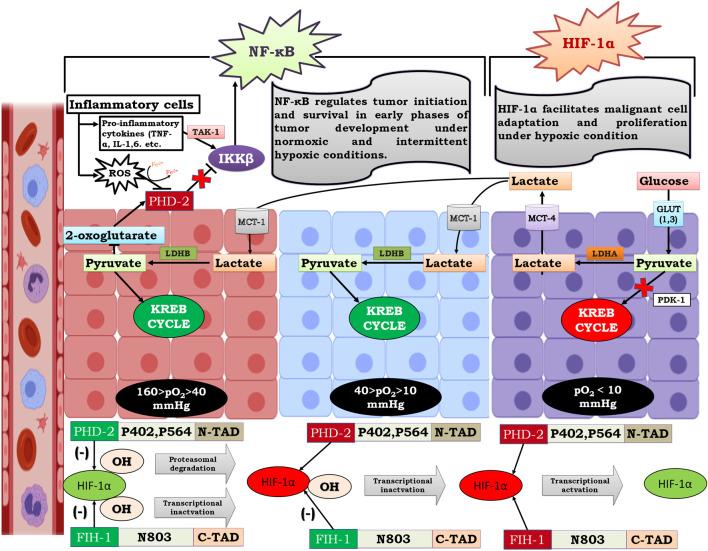

## 1 Introduction

**FIGURE 1:**
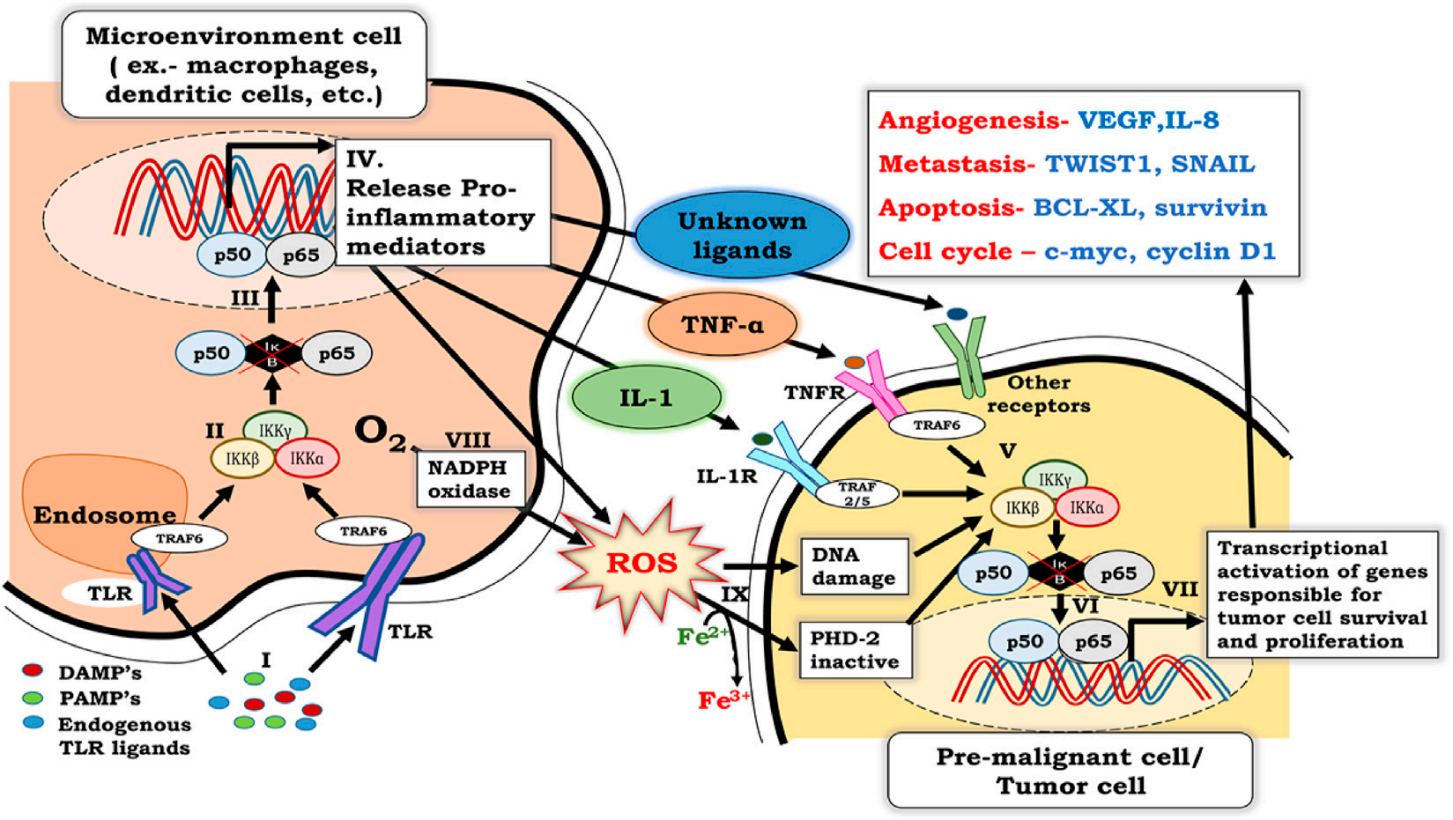
Crosstalk between NF-κB signaling in inflammatory cells and premalignant cells. I. NF-κB activation in inflammatory cells involves binding of PAMPs, DAMPs, and endogenous TLR ligands with TLR receptors, which are associated with TRAF-6. II. Binding of PAMPs, DAMPs, and endogenous TLR ligands with TLR’s leads to activation of the IKKαβγ complex, which further results in phosphorylation, ubiquitination, and degradation of IκB. III. Degradation of IκB renders p50 and p65 heterodimers free to migrate to the nucleus and activate NF-κB. IV. Activation of NF-κB in inflammatory cells results in the secretion of pro-inflammatory cytokines, including TNF-α and IL- 1. V. Released pro-inflammatory cytokines bind to their specific receptors on pre-malignant cells and activate the IKKαβγ complex, which further results in phosphorylation, ubiquitination, and degradation of IκB. VI. Degradation of IκB renders p50 and p65 heterodimer free to migrate to the nucleus and activates the transcription of genes responsible for cell survival, proliferation, migration, metastasis, and angiogenesis. VII. ROS generated by phagocytic NADPH to counter infectious pathogens, along with the ROS generated by activation of NF-κB in inflammatory cells, enters the cytosol. VIII. Oxidative stress caused by large amounts of ROS not only damages DNA, but also ionizes Fe^2+^ to Fe^3+^. As a result, PHD-2, which requires Fe^2+^ as a cofactor for its activity, is inactivated. ROS-induced DNA damage and PHD-2 inactivation further contribute to NF-κB activation in pre-malignant cells.

Cancer is a genetic abnormality in which the body’s old or flawed cells evade signals of programmed cell death and acquire uncontrolled replicative potential. Cancer can be categorized as solid tumors (carcinoma, sarcoma, melanoma, and lymphoma) and leukemia (no cell mass formation). However, all genetic aberrations that result in abnormal cell growth is not cancer. Mutation in cells results in the formation of “neoplasm”.The neoplasm may either bud into an enormous cell mass that is not malignant as well as remains confined to a particular location is referred to as a “benign tumor” (like adenomas, fibroids, hemangiomas and lipomas) or may possess malignant characteristics and metastasize to nearby organs and tissues through blood and lymph referred to as a “malignant tumour” (like adenocarcinomas, basal cell carcinomas and squamous cell carcinomas) (Cooper and Hausman, 2007); Fares et al., 2020). Although there are several causes of cancer, inflammation remains a significant one. An essential contributor to the formation of malignant tumours is an exaggerated immune response to inflammation that results in a condition known as chronic inflammation (Karin et al., 2006; Schottenfeld and Beebe-Dimmer, 2006). TNF-, IL-1, IL-7, IL-8, IL-17, and other pro-tumorigenic cytokines and interleukins are secreted by infiltrating immune cells at the site of infection in a chronic inflammatory microenvironment (León et al.; Hung et al., 2012; Fu et al., 2015; Hospital, 2018; Seol et al., 2019).In combination with reactive oxygen species (ROS), these pro-tumorigenic cytokines lead to DNA damage by inducing genotypic changes in degraded tissue mucosa in favour of tumour initiation and development (Kryston et al., 2011; Kidane et al., 2014) (Figure 1). In the initial phase of tumor growth, oxygen supply is not a limiting factor for mutant cell survival because of easy access to pO2 from nearby vasculature. As the size of the tumor increases to more than 400µm, a hypoxic environment is created, especially at the center of cancer, because nutrients and oxygen can diffuse only up to a radius of 200 µm (Christensen et al., 2010; Grimes et al., 2014; Däster et al., 2017; Riffle and Hegde, 2017). In this regard, the tumor mass can be divided into three distinct zones: the non-hypoxic zone, intermittent hypoxic zone (OXOPHOS), and severe hypoxic zone. Bioenergetics in these three zones is highly interdependent and complex. Early transmuted (non-hypoxic) cells that make up the lining of blood vessels aerobically derive energy from glucose through glycolysis and oxidative phosphorylation. However, in fast-proliferating cells, O_2_ is a limiting factor for oxidative phosphorylation.

Consequently, glycolysis is the only source of energy in rapidly proliferating tumor cells, which is evident from the increased intracellular accumulation of glycolysis’s end product, pyruvate. Accumulated pyruvate is converted into lactate in hypoxic cells. The lactate generated is transferred into non-hypoxic and OXOPHOS cells through monocarboxylate transporter-1 (MCT-1). Hypoxic cells that form the core of the tumor mass are under the control of hypoxia-inducible factor -1α (HIF-1α). The stabilization and transcriptional activity of HIF-1α is controlled by two oxygen-dependent dioxygenases, namely, prolyl hydroxylase-2 (PHD-2) and factor inhibiting hypoxia-inducible factor-1 (FIH-1), through the N-terminal and C-terminal domains, respectively (Qutub and Popel, 2006; Brahimi-Horn and Pouysségur, 2007; Hu et al., 2007; Koh and Powis, 2012; Akanji et al., 2019).It would be appropriate to mention that previous studies have pointed out differential regulation of HIF-1α by NTAD and CTAD (Dayan et al., 2009); only CTAD is reported to directly bind with the co-activators CBP/P300 through the CH-1 region and regulate HIF-1α transcriptional activity. In contrast to CTAD, the mechanism that regulates the transcriptional function of NTAD is poorly understood. Moreover, it has also been reported that NTAD is responsible for gene specificity and regulates proteasomal degradation rather than transcriptional activation of HIF-1α (Dayan et al., 2006; Hu et al., 2007).

Studies have shown that as the oxygen gradient in solid tumors falls from 40 mmHg pO2 (5% O_2_) in the non-hypoxic zone to 10 mmHg pO_2_ (1% O_2_) in the intermittent hypoxic (OXOPHOS) zone, PHD-2 loses its activity, whereas FIH-1 is inhibited only under severely hypoxic conditions <10 mmHg pO_2_ (1% O_2_) (Dayan et al., 2006; 2009). Thus, in the non-hypoxic zone where PHD-2 and FIH-1 are functional, HIF-1α is degraded and transcriptionally inactivated. In contrast, in the OXOPHOS zone, HIF-1α is stabilized due to the inactivation of PHDs but remains transcriptionally inactive due to the presence of FIH. In the hypoxic zone, HIF-1α is both stable and transcriptionally active due to the inactivation of both PHDs and FIH-1. As the O_2_ concentration diminishes in the intermittent hypoxic and severely hypoxic zone during tumor development, HIF-1α becomes stabilized and progressively becomes transcriptionally active due to the inactivation of PHD first and FIH-1 second. In such cases, angiogenesis, glycolysis, fatty acid synthesis, migration, metastasis, malignant cell survival, and proliferation are regulated in non-hypoxic and intermittent hypoxic zones to support tumor progression. There is an intriguing possibility that during the early stage of tumor initiation and progression when oxygen tension is limiting for transcriptional activity HIF- 1α, other mechanisms could be responsible for regulating malignant cell survival and proliferation.

Research over the past decade has pointed out that NF-κB is a driver of inflammation that gives rise to cancer under a chronic inflammatory microenvironment (Pikarsky et al., 2004). Activation of NF-κB in both premalignant cells and cells of the microenvironment (phagocytes, T cells, and B cells) is crucial in the early stages of tumor development and progression (Wang et al., 2014). NF-κB is activated firstly in cells of tumor microenvironment in response to binding of pathogen-associated microbial patterns (PAMPs) and danger-associated molecular patterns (DAMPs) with toll-like receptors (TLRs). This association eventually activates the IKK complex, leading to transcriptional activation of NF-κB in cells of the microenvironment. In response to NF-κB actuation, cells in the tumor microenvironment secrete proinflammatory mediators, such as TNF-α and IL-1. These proinflammatory mediators act on their specific receptors present on premalignant cells and further lead to the induction of NF-κB in premalignant cells through the IKKβ-mediated canonical NF-κB signaling pathway (Martins et al., 2016). This canonical stimulation of NF-κB induces genes involved in cellular proliferation, survival, angiogenesis, and metastasis.

In this study, we propose to determine the possible role of lactate shuttle in the activation of NF-κB in non-hypoxic and intermittent hypoxic malignant cells. We have also directed our study to answer few specific questions.

a) How, NF-kappa B and HIF-1α play a crucial role in tumorigenesis and progression.
b) To what extent does factor inhibiting HIF-1α (FIH1) continue to function while Prolylhydroxylase-2 (PHD-2) activity is suppressed by the lactate shuttle in non-hypoxic and intermittently hypoxic cells?
c) Under normoxia, NF-κB is activated at the expense of HIF-1α due to the downregulation of PHD2 mediated by the lactate shuttle.
d) In the early stages of carcinogenesis, when oxygen tension is normal, HIF-1α is inactive. How can NF-κB promote tumour initiation and survival during this time?
e) PHD2 mediates NF-κB and HIF-1α double regulation, suggesting it may be a useful novel pharmacological target.

## 2 How inflammation contributes to the formation of neoplasm?

### 2.1 Inflammation

Inflammation is the first line of the body’s defense mechanism that protects against infection and injury. Inflammation is defined by a sequence of responses involving vasodilation, migration of immune cells, and leakage of plasma proteins at the site of disease or injury. Phagocytic cells that arrive at the site of inflammation, especially neutrophils, macrophages, and dendritic cells, express pattern recognition receptors called “Toll-Like Receptors” (TLR’s). The binding of inflammatory factors (such as cytokines, chemokines, PAMPs, and DAMPs) with TLRs triggers a signaling cascade that leads to the induction of NF-κB (Freedman et al., 2002; Tolle and Standiford, 2013; Chen et al., 2018). NF-κB is a primary transcriptional regulator of inflammation and regulates tissue repair and wound healing (Landén et al., 2016).

The inflammatory response to an infection or injury usually subsides after tissue repair; however, an exaggerated inflammatory response results in a condition known as chronic inflammation. During chronic inflammation, leaky vasculature and increased ATP requirement cause hypoxia. Under such a hypoxic inflammatory microenvironment, infiltrating immune cells at the site of infection or injury produce reactive oxygen species (ROS), which causes damage to the DNA by altering gene expression and genetic sequences. The accumulation of ROS and cytokines further elevates NF-κB activity to create a pro-tumorigenic microenvironment that favors tumor initiation and development (Liou and Storz, 2010). Thus, the NF-κB pathway mediates the crosstalk between chronic inflammation and cancer, in which early transmuted malignant cells proliferate and form tiny tumors (Ben-Neriah and Karin, 2011).

### 2.2 NF-κB regulatory machinery

NF-κB has been reported to govern the expression of the immunoglobulin κ-light chain in B lymphocytes. Later, NF-κB was recorded as a group of transcription regulators consisting of NF-κBp65 (RelA), RelB, Rel-c, (p50/p105), and (p52/p100) subunits (Sen and Baltimore, 1986). These transcription factors comprise a preserved Rel homology domain (RHD) that facilitates them to undergo homo or hetero-dimerization (Perkins, 2012).The major heterodimer of NF-κB is p65/50, which remains segregated in the cytoplasm and tightly clubbed with an inhibitor of kappa B (IκB) family proteins (comprising IκBα, IκBβ, and IκBε) along with two precursors, that is, p105/IκBγ and p100/IκBδ) (Ghosh et al., 1995). The binding of NF-κB dimers to IκBs with the help of 7–8 ankyrin repeats (Malek et al., 2003) prevents nuclear translocation of NF-κB dimers and consequently inhibits their transcriptional activity (Marienfeld et al., 2003).

Pathways regulating NF-κB transcriptional activity include the canonical pathway (or classical pathway) and the alternative pathway (or the non-canonical pathway) (Sun, 2011; Sun et al., 2013; Dorrington and Fraser, 2019). The key regulator of these pathways is a cytoplasmic complex known as the “IKK complex.” The IKK complex is composed of the catalytic subunits IKKα (or IKK1) and IKKβ (or IKK2) along with the modulatory subunit IKKγ (or NEMO). Both IKKα and IKKβ share 52% sequence chronology and 70% homology but play distinct yet pivotal roles in pan NF-κB regulation (Liu et al., 2012). NEMO/IKKγ has an N-terminal coiled-coil domain that interacts with IKKα and IKKβ and mainly functions as a regulatory subunit in the IKK complex. IKKα and IKKβ are essential regulators of IκBs, and their activation is necessary for all NF-κB signaling pathways, whether classical or alternative (Oeckinghaus et al., 2011). Release of NF-κBp65 dimer from the inhibitor of kappa B (IκB) requires phosphorylation of IKKα or IKKβ at specific serine residues in the activation loop, that is, serine-176(S-176) and serine-180(S-180) for IKKα and serine-177(S-177), and serine-181(S-181) for IKKβ. Once activated, IKKα or IKKβ, in turn, phosphorylates inhibitor of kappa B (IκBs), further leading to β-TrCP-dependent E3 ubiquitin ligase-mediated 26s-proteasomal degradation of inhibitor of kappa B (IκBs). Henceforth, the NF-κB dimer can migrate from the cytoplasmic space to the nucleus, triggering gene expression (Gilmore, 2006; Dorrington and Fraser, 2019).

#### 2.2.1 IκB kinase (IKK) function

The pivotal step in NF-κB induction is cytokine-inducible phosphorylation specific for amino-terminal regulatory serines at Ser32 and Ser 36 of IκBα or at Ser19 and Ser 23 in IκBβ. Even though various enzymes mediate phosphorylation of IκB, only IKK meets the acrostics of IκBα/β degradation, which includes fast signal induction and concurrent phosphorylation of the pair of serine residues, Ser32 together with Ser 36 of IκBα and Ser19 along with Ser 23 in IκBβ, respectively (Gilmore, 2006).

Another essential characteristic of IKK-mediated phosphorylation is the choice of serine as a target for phosphorylation over thionine, which matches IκBα/β degradation. As discussed previously, IKKα and IKKβ are catalytic subunits of the IKK complex, which are stimulated in cells in response to TNF-α and IL-1. Both subunits possess an activation loop in their kinase domains, similar to other protein kinases, along with a sequence homology between both kinases (Johnson et al., 1996; Mercurio et al., 1997). The activation loop contains specific sites on IKKα (S176, S180) and IKKβ (S177, S181), the phosphorylation of which results in a conformational change that is responsible for kinase activation (Ling et al., 1998). Moreover, replacing serine with alanine inhibits the activation of IKK, although a similar substitution with glutamic acid imitates activity equal to phosphoserine. It would be appropriate to mention that modification in S176 and S180 of IKKα to alanine, either on one or both of the serine residues, does not have any effect on TNF-α-and IL-1 mediated IKK activity (Mercurio et al., 1997; Delhase, 1999). This finding suggests that, in the case of proinflammatory stimuli, phosphorylation of IKKα is not necessary for stimulation of the IKK complex. Research has also indicated that similar modifications of S177 and S181 in IKKβ to alanine abolished TNF-α-and IL-1 mediated IKK activity. Thus, activation of the IKK complex by proinflammatory stimuli depends entirely on the phosphorylation of IKKβ and not on IKKα.

#### 2.2.2 The canonical pathway (classical pathway)

**FIGURE 2:**
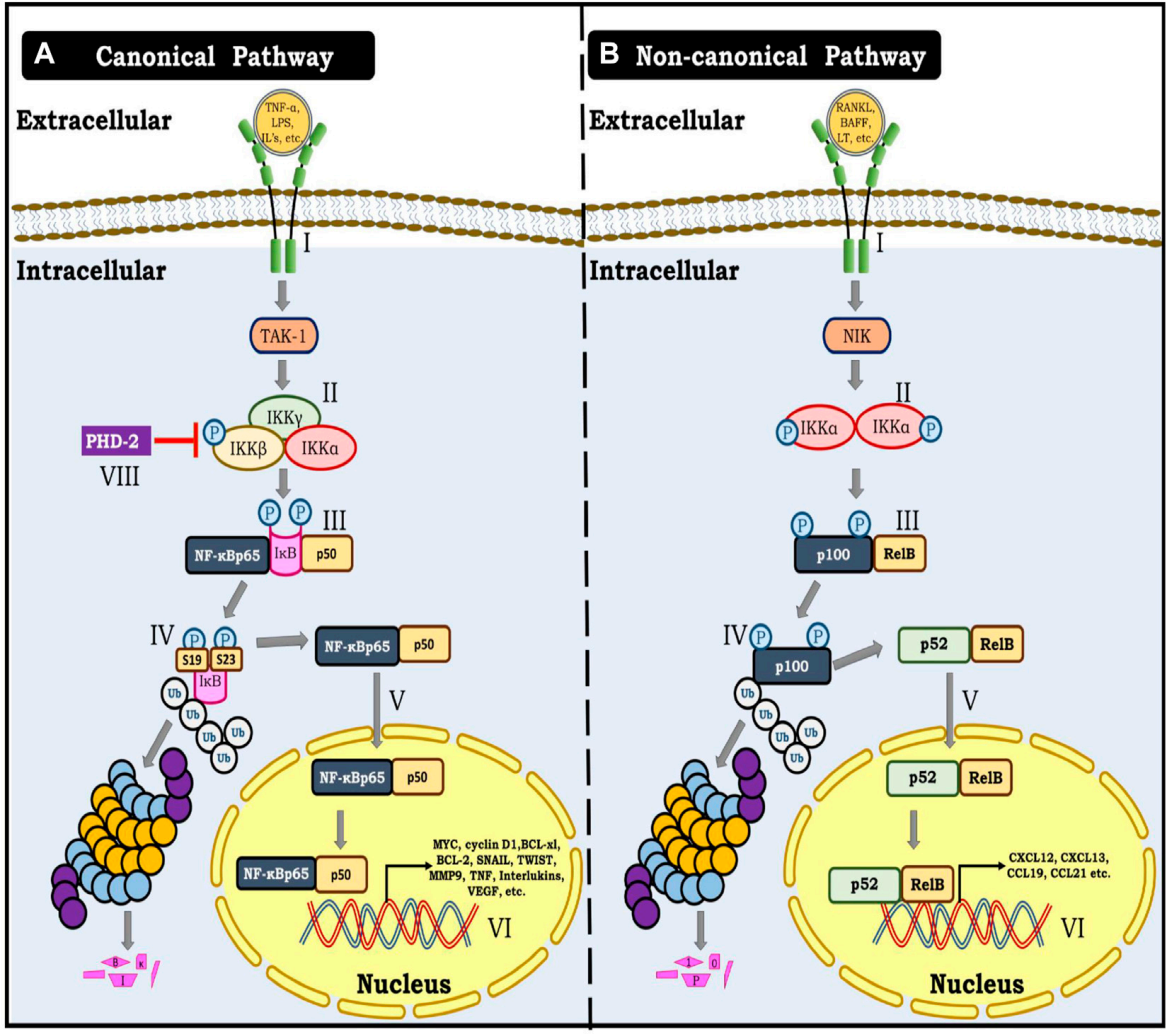
**(A)** Canonical NF-κB pathway. I- Represent binding of TNF-α, Interleukins, Lipopolysaccharides and other cytokines with their specific receptor resulting in activation of TAK kinase-1. II- Activation of TAK-1 result in phosphorylation of IKKΒ unit of the triad consisting of IKKα, IKKβ and IKKγ. III- Phosphorylation of IKKΒ further result in phosphorylation of IκB at Ser19 and Ser23. IV- This phosphorylation allows for ubiquitination and proteasomal degradation of IκB. V- This modification renders NF-κB dimer (p65 and p50) free to translocate to the nucleus. VI- NF-κB dimer (p65 and p50) further binds with specific response elements on DNA and triggers gene transcription of several genes involved in angiogenesis, metastasis, migration and cellular proliferation. **(B)** Non- canonical NF-κB pathway. I- denotes the binding of TNFR ligands to their respective receptors, which results in the activation of NF-κB inducing kinase-1 (NIK). II- NIK phosphorylates IKKα, causing it to be activated. III- Finally, activated IKKα phosphorylates p100. IV- IKKα mediated phosphorylation of p100 marks it for ubiquitination and proteasomal processing to yield p52. V-p52 forms an active dimer with Rel B and translocates to the nucleus. VI- p52 and Rel B dimer bind to the DNA and cause gene transcription.

The canonical pathway is activated by the binding of TNF-α, interleukins, and chemokines to their specific receptors on cell membranes, which starts the IKK complex. The exact mechanism and the proteins involved in the activation of this complex are under investigation. However, stimulation by proinflammatory cytokines such as Ilβ1, TNF-α, and TLR ligands in the case of toll-like receptors (TLR) in macrophages and mouse embryonic fibroblasts (MEFs), triggers TGF-β-activated kinase 1 (TAK-1)-mediated phosphorylation of IKKβ at S177, which subsequently catalyzes autophosphorylation at S181, resulting in activation of the IKKβ complex. Furthermore, this activated cytoplasmic complex phosphorylates IκBα at serine residues S32 and S36, whereas for IκBβ at serine residues S19 and S23. This phosphorylation primes IκBs for ubiquitination by the (SCF)/(β-TCP) E3 ubiquitin ligase complex, followed by proteasomal degradation, which facilitates the release of NF-κB dimers. NF-κB dimers, released, are free to migrate to the nucleus and trigger transcription of genes involved in various cellular functions and bioenergetics, including cellular proliferation and metabolism (Gilmore, 2006; Oeckinghaus et al., 2011) (Figure 2).

#### 2.2.3 The non-canonical or alternative pathway

Unlike the canonical NF-KB pathway, which is fast and activated by a wide range of inflammatory cytokines and other external factors, the non-canonical pathway is slow and only activated by a small subset of TNF-superfamily receptors, such as LTβR, BAFFR, CD40, CD30, CD27, RANK, TNFR2, FN14, and CD134. Henceforth, the biological functions of this pathway are more specialized and specifically associated with lymphoid organ and immune cell development, as well as immunological response and homeostasis (Sun, 2017).

Briefly, the non-canonical NF-κB pathway is activated by NF-κB inducing kinase (NIK). In the inactive state, NIK remains bound to TRAF3 which is complexed with TRAF2 and cIAP1/2. Further, cIAP1/2 ubiquitinates NIK, marking it for proteasomal degradation. However, binding of TNFR ligands (such as BAFF, RANKL, LTα1β2, TWEAK, etc.) with their specific receptors, TRAF3, TRAF2, and cIAP1/2 triad complex is recruited to the membrane binding site of the receptor-ligand complex, leaving NIK unbound. Moreover, cIAP1/2, instead of ubiquitinating NIK now ubiquitinates TRAF3 and marks it for proteasomal degradation which further facilitates the stabilization of NIK. As a result, NIK starts accumulating, and its level inside the cell increases. Consequently, NIK phosphorylates and induces IKKα homodimer. Activated IKKα in turn phosphorylates C-terminal serine residues (866 and 870) of the p100 subunit of inactive RelB and p100 heterodimer. The subsequent transduction mechanism involves p100 ubiquitination by the (SCF)/(β-TCP) E3 ubiquitin ligase complex followed by proteasomal processing to yield a p52 subunit. This event is followed by the association of p52 with RelB to form an active dimer which translocates to the nucleus and triggers gene transcription (Figure 2) (Sun, 2011).

Solid tumors primarily exhibit canonical signaling, whereas hematological cancers primarily exhibit non-canonical signaling (Kaltschmidt et al., 2018). Therefore, canonical signaling is the main subject of this review.

### 2.3 Biological effects of NF-κB in cancer cells

#### 2.3.1 Effect of NF-κB on cell cycle

NF-κB is the critical modulator of the tumor microenvironment in the early stages of tumor development. NF-κB is integral to tumorigenesis, involving tumor initiation (formation of transformed cells), tumor promotion (early transformed cells proliferate rapidly and increase in both size and number), and tumor progression (transformed cells acquire malignant potential) (Pitot et al., 1981; Barcellos-Hoff et al., 2013). It takes approximately three to five oncogenic mutations for a normal cell to become malignant. This is because most oncogenic mutations are acquired *de novo*, and rarely, germline mutations result in cancer (Fearon and Vogelstein, 1990; Grivennikov et al., 2010).

Under non-hypoxic or intermittent hypoxic conditions, wherein HIF-1α is dormant, NF-κB initiates tumor growth by enhancing reactive nitrogen species and ROS generation that cause damage and impart oncogenic characteristics. Moreover, NF-κB activation results in cell cycle alterations and ensures that both DNA strands acquire mutations and transfer to progenitor cells (Ledoux and Perkins, 2014; Kiraly et al., 2015). NF-κB may also cause aneuploidy and epigenetic changes, resulting in tumorigenesis (Ren et al., 2011; Nakshatri et al., 2015). Further, by inhibiting p53 induced cell death, NF-κB activation favors the malignant transformation of DNA damaged cells (Gudkov et al., 2011). Henceforth, in solid tumors, NF-κB regulates the transformation of normal cells to premalignant cells and other premalignant cells to malignant cells by inhibiting p53 induced programmed cell death and enhancing the production of ROS and reactive nitrogen species.

#### 2.3.2 Effect of NF-κB on programmed cell death

NF-κB suppresses apoptosis and enhances cellular proliferation in various cancer pathologies, including B-cell lymphoma, neuroblastoma, and breast carcinoma (Smith et al., 2014; Zhi et al., 2014; Knies et al., 2015). A common determinant among various tumors is the intrinsic NF-κB activity that confabs intransigence to cell death by up-regulating anti-apoptotic genes. Moreover, in the tumor microenvironment, NF-κB operates in a paracrine fashion to expedite tumor cell proliferation (Greten et al., 2004; Bassères and Baldwin, 2006). To repress apoptosis, NF-κB activates a group of targeted genotypes that impede distinct steps of the extrinsic and intrinsic apoptotic pathways. The targets of NF-κB-mediated suppression of apoptosis include inhibitors of apoptotic proteins such as XIAP, c-IAP1, and c-IAP2. It is relevant to mention that XIAP, c-IAP1, and c-IAP2 are involved in the inhibition of pro-caspase-9 and blockade of caspase-3 and caspase-7 activity. Among other targeted genes, BCL-2, BCL-XL, and NR13, which belong to the BCL-2 family (Liston et al., 2003; Fulda, 2014). Therefore, NF-κB has a pivotal role in tumor cell survival by activating IAP’s and BCL-2 family proteins. Therefore, by activation of inhibitors of apoptotic proteins and BCL-2 family proteins, NF-κB plays a pivotal role in malignant cell survival in solid tumors.

#### 2.3.3 Effect of NF-κB on angiogenesis

Inflammation, as well as in an NF-κB dependent manner, stimulates angiogenesis. Research has shown that constitutive NF-κB activity regulates IL-8 and VEGF activity in cancer cells (Huang et al., 2000; Bonavia et al., 2012). Furthermore, NF-κB, in conjunction with iNOS, stimulates the production of proteases and NO, which has a pivotal function in inflammation-induced angiogenesis (Zhang et al., 2005; Costa et al., 2007). In addition, NF-κB-targeted genes such as fibroblast growth factor, IL-8, matrix metalloproteinase-9 (MMP-9), and others are involved in various steps in the regulation of angiogenesis (Zhang et al., 2005). It is pertinent to mention that MMP-2, 3, and 9 play an integral role in the breakdown of the basement membrane as well as in remodeling of the extracellular matrix, which not only facilitates cell migration but also favors either angiogenesis (endothelial cells) or metastasis (malignant cells) depending on the microenvironment (Huber et al., 2004). Furthermore, in the tumor microenvironment, cancer-associated fibroblasts (CAFs) facilitate the deposition of collagen and another extracellular matrix (ECM) components. CAFs are activated partially in an NF-κB-dependent manner and are vital in the actuation of proinflammatory genes that regulate TNF-α, interleukin (IL-1β and IL-6, VEGF, CXCL2, SDF-1, and many other chemokines, thereby enhancing angiogenesis (Disis, 2010; Erez et al., 2010; Kalluri, 2016). The above findings suggest that NF-κB, through stimulation of iNOS, cytokines, chemokines, and CAF in the tumor microenvironment, facilitates new vessel formation to support budding tumors.

#### 2.3.4 NF-κB in invasion and metastasis

The key factor contributing to invasion in the case of solid tumors is acidosis. To survive under non-hypoxic and intermittent hypoxic conditions malignant cells depend upon glycolysis despite the presence of O2, a phenomenon called the “Warburg Effect” (Warburg, 1956; Chandel, 2021). Whereas, under hypoxic conditions malignant cells utilize anaerobic glycolysis for energy production, a phenomenon called the “Pasteur Effect” (Kim et al., 2006; Milane et al., 2011). However, the end product of glycolysis, in both the cases, is lactic acid, resulting in the accumulation of lactic acid inside the cell (Gatenby et al., 2007). To survive, efflux of lactic acid from the cell by MCT-4 is necessary. The resultant increase in acidity and decrease in pH of the extracellular environment generates ROS and facilitates the activation of NF-κB (Gatenby and Gillies, 2004; Gupta et al., 2014).

NF-κB can directly trigger the expression of genes that stimulate epithelial to mesenchymal transition (EMT) and offer invasive characteristics to malignant cells, such as TWIST1, SLUG, and SNAIL (Wu and Zhou, 2009; Pires et al., 2017). Stimulation of these genes initiates neoangiogenesis and promotes EMT-induced cancer cell extravasation into the blood and lymphatic vessels. Therefore, preparing a protective pre-metastatic niche that allows for the survival and proliferation of metastatic initiating tumor cells (Psaila and Lyden, 2009).

Other mechanisms by which NF-κB promotes migration and metastasis is through upregulation of proto-oncogenes such as c-myc and cyclin D1 (You et al., 2002; Ledoux and Perkins, 2014), regulating selectins and integrins, which are essential players in invasion and colonization at distal sites (Collins et al., 1995; Nguyen et al., 2009), and increasing cell surface expression of the chemokine receptor CXCR4, thereby promoting metastasis and invasion (Helbig et al., 2003). From the above discussion, it is clear that lactic acidosis is a prominent regulator of NF-κB in solid tumors, which provides cells with invasive potential and protects them with a pre-metastatic niche.

Till now, we have seen that hypoxia arising from an exaggerated immune response to inflammation activates NF-κB, which regulates downstream mechanisms of tumor initiation and development. Now, as the tumor size keeps on increasing, as it increases more than 400 μm, cells at the center of the tumor are under severely hypoxic conditions. To survive under such a hostile environment, these cells take the help of another transcription regulator that we will see in the following section of this manuscript.

## 3 How do tumor cells adapt under hypoxia?

### 3.1 Hypoxia

Perturbation in oxygen supply results in a condition termed hypoxia, characterized by reduced pO_2_ in a particular tissue compared to pO_2_ in well-vasculature tissues (Semenza, 2014). Tumor hypoxia results from either reduced oxygen delivery due to occlusion or leakage of blood vessels or increased oxygen consumption, which may be attributed to rapid cellular division (Vaupel et al., 2004).

### 3.2 Mechanistic regulation of HIF-1α

One of the significant factors contributing to the adaptation of malignant cells to the hypoxic tumor environment is the hypoxia-inducible factor (HIF) (Vaupel et al., 2004; Semenza, 2012). The master transcriptional regulator HIF belongs to a group of two bHLH domain-containing proteins from the PAS (PER-ARNT-SIM) family (Wang et al., 1995), with three isoforms: HIF-1, 2, and 3 (Wang and Semenza, 1995; Gu et al., 1998; Keith et al., 2012). Each isoform of HIF has two subunits, α, and β. The α-subunit is present in the cytoplasm and is regulated by hypoxia. The aryl hydrocarbon receptor nuclear translocator (ARNT), commonly referred to as the β-subunit, is integrally expressed in the nucleus and is induced by dimerization with the α-subunit (Jiang et al., 1996; Déry et al., 2005). HIF-1α acts as an oxygen sensor and is regulated by two sets of enzymes belonging to a class of 2- oxoglutarate, ascorbate, and iron-dependent dioxygenases called prolyl hydroxylases (PHDs) and factor inhibiting hypoxia-inducible factor-1 (FIH-1). Prolyl hydroxylase exists in three isoforms: PHD-1, 2, and 3. Among all PHDs, PHD-2 is the most prominent regulator of HIF-1α in solid tumors (Kaelin and Ratcliffe, 2008)].

**FIGURE 3:**
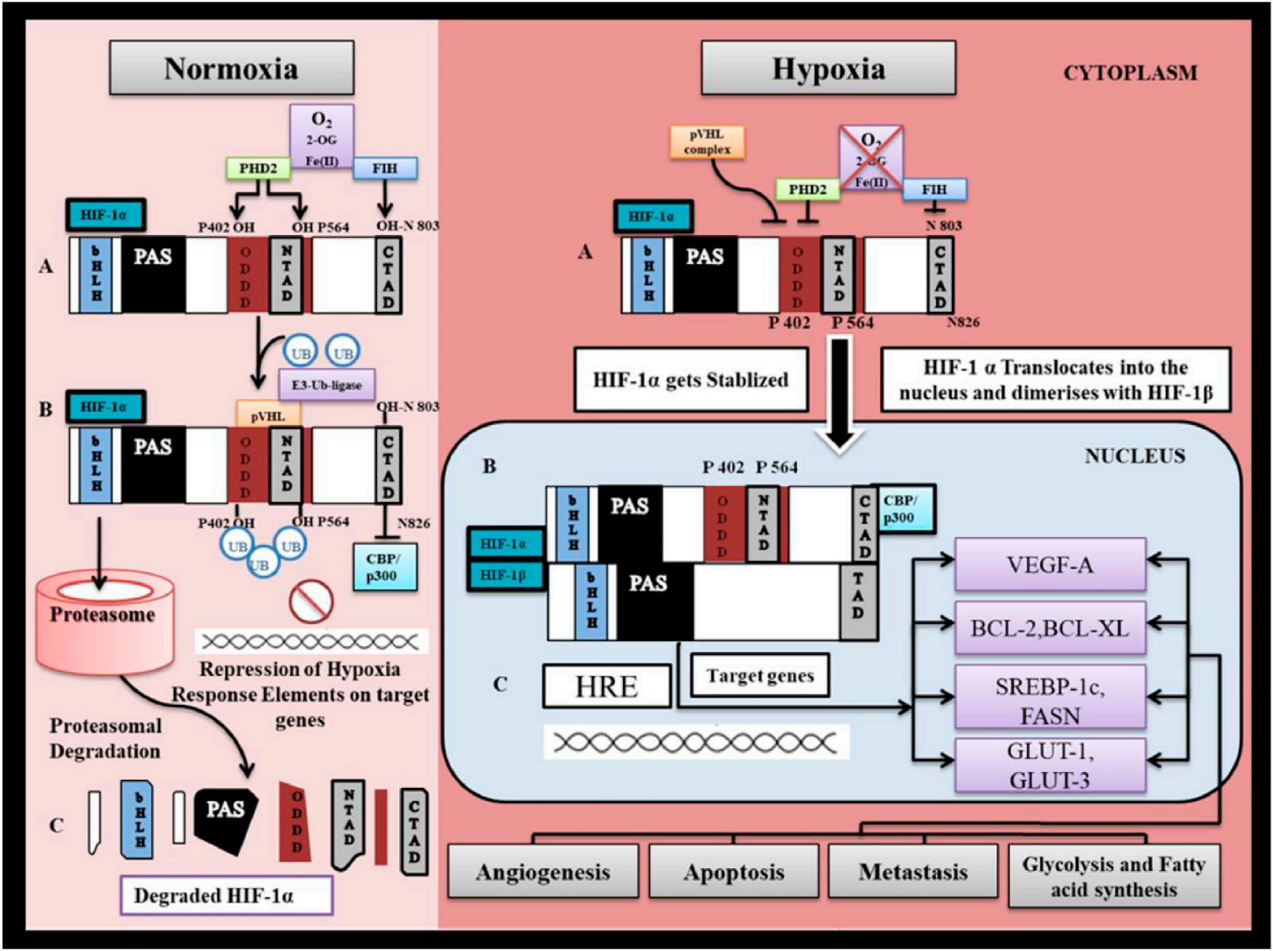
HIF-1α regulation under normoxia and hypoxia. I. Normoxia represents pO_2_ > 40 mm Hg. (A) Under normal oxygen tension, PHD2 (prolylhydroxylase-2) hydroxylates HIF-1α at proline residues P402 and P564 and Factor Inhibiting HIF-1α hydroxylates HIF-1α at asparagine residue N803. in presence of O2 as substrate, Oxoglutarate as co-substrate and Fe as cofactor. (B) Prolyl Hydroxylation of HIF-1α allows for binding of pVHL to NTAD ( N-terminal transactivation domain) of HIF-1α. Binding of pVHL recruits ubiquitinin by activation of E3- ubiquitinin ligase which marks HIF-1α for proteasomal degradation. (C) HIF-1α is degraded by 26S proteasome thus preventing stabilization and accumulation of HIF-1α. Asparginyl hydroxylation of HIF-1α at CTAD (C-terminal transactivation domain) prevents binding of CBP/P300 to the CTAD (CH- 1 domain) and thereby inhibiting downstream mechanism involved in HIF-1α transactivation. II. Hypoxia represents pO_2_ < 40 mmHg. (A) Under hypoxia HIF-1α escapes degradation and gets stabilized since PHD2 enzymes which are responsible for its proteasomal degradation are inhibited. (B) Stabilized HIF-1α gets accumulated in the cytosol till the oxygen tension reaches the level (pO_2_> 10 mmHg) that it becomes limiting for FIH-1, such that CBP/P300 proteins are able to bind to CTAD. (C) HIF-1α translocate into the nucleus to bind with β-subunit that is constitutively expressed in the nucleus. HIF-1α further binds with hypoxia response element on targeted gene and trigger gene transcription.

Under non-hypoxic conditions (pO_2_ > 40 mmHg) (Figures 3), HIF-1α undergoes proteasomal degradation by PHD-2 *via* a process that involves post-translational hydroxylation of HIF-1α at proline residues (P402 and P564) in the human sequence present in O2 dependent degradation (ODD) domain (Bruick, 2001; Schofield and Ratcliffe, 2005). Hydroxylation eventually allows the binding of von Hippel-Lindau tumor suppressor protein (pVHL) and recruitment of E3-ubiquitin ligase that combine to form the E3-ubiquitin ligase complex, which ultimately marks HIF- 1α for degradation by the 26-S proteasome (Maxwell et al., 1999; Kaelin, 2003). On the other hand, FIH hydroxylates HIF-1α at an asparagine residue (N803) that abrogates the binding of histone acetyltransferases such as P300 and CBP (CREB binding protein (CBP) to the C-terminal transactivation domain (CTAD) on HIF-1α and blocks the transactivation of genes regulated by HIF-1α (Lando et al., 2002; McNeill et al., 2002).

Under hypoxic conditions such as ischemia and cancer, as the oxygen tension (pO_2_) reaches below 40 mmHg, PHD2 is inactivated, followed by FIH (at pO_2_ < 10 mmHg). In the pO_2_ range of 40 and 10 mmHg, due to inactive PHD-2, HIF-1α escapes proteasomal degradation and subsequently stabilizes, and its cytoplasmic level increases. However, at this stage, the transcriptional function of HIF-1α is inhibited by FIH-1 activity (Koivunen et al., 2004; Stolze et al., 2004; Rani et al., 2022)^.^ As pO_2_ deteriorates further and reaches below <10 mmHg, FIH loses its functional activity (Dayan et al., 2006). After that, cytoplasmic HIF-1α migrates to the nucleus, where it forms a dimer with HIF-1β. The HIF-1αβ dimer further binds with co-factor CBP/P300 to interact with HIF-1α responsive element “5′-RCGTG-3′” on target genes and triggers gene transcription (Semenza et al., 1996).

**FIGURE 4:**
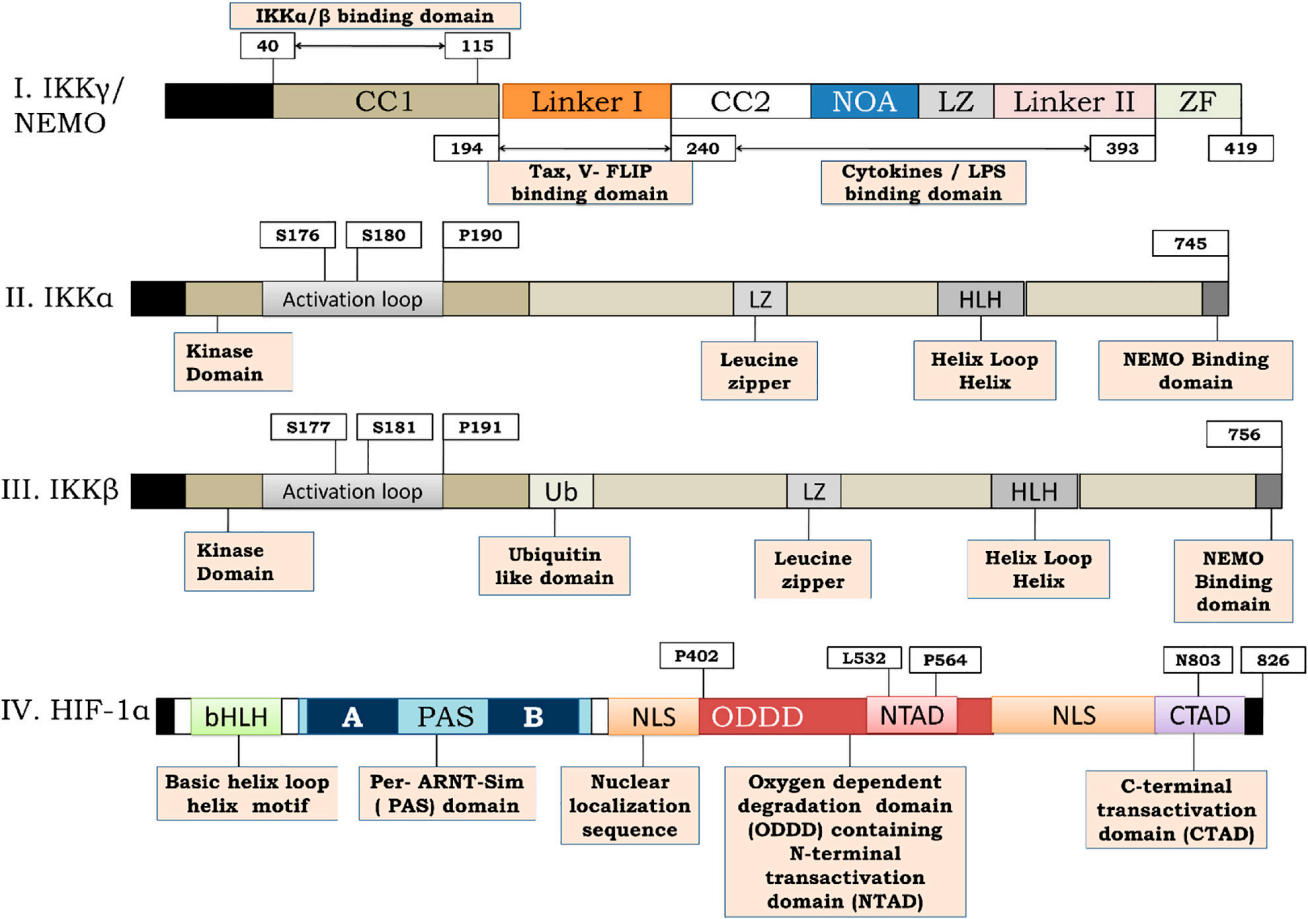
Structure of IKKγ, IKKα, IKKβ and HIF-1α. **(I)** Represents the structure of IKKγ or NEMO. IKKγ is a 419 amino acid dimeric fragment consisting of a series of parallel intermolecular coiled coil domains (represented as CC1 and CC2). The amino acid terminus (N-terminus) is vital for interaction with IKKα and IKKβ. Linker 1 serves for interaction with viral trans activators like HTLV-1 and Tax and V-Flip. C-terminus function for signal transmission while NOA and Zinc Finger (ZF) domains bind to polyubiquitin chains. **(II,III)** represents the structure of IKKα and IKKβ respectively. IKKα is a 745 amino acid fragment consisting of an activation loop from amino acid 176–180 whereas IKKβ is a 756 amino acid fragment consisting of activation loop from 177–181 on amino terminous. A ubiquitin like domain is present (from amino acid 307–384) on carboxy terminous of IKKβ but not on IKKα. The function of leucine zipper domain is to allow homo and heterodimerization of the kinases. The function of helix loop helix is less clear but it seems to be involved in the modulation of kinase activity. A 40 amino acid region at the extreme carboxy terminous of the kinases (AA-705–743) is required for their interaction with NEMO. **(IV)**- represents the structure of HIF-1α. HIF-1α is a 826 amino acid sequence consisting of basic helix loop helix motif and a PAS domain. The sequence possess a N-terminal trans activation domain (N-TAD) on Oxygen dependent degradation domain (ODDD) of HIF-1α along with C-terminal transactivation. The HIF-1α consists of prolyl hydroxylation site at Pro402 and P4 as w ro56ell as pVHL binding site at Leu532 respectively on that lie on ODDD. The CTAD of HIF-1α consists of Asparginyl hydroxylation site at N803.

Apart from HIF-1α, hypoxia also activates NF-κB through phosphorylation of IKKβ (Koong et al., 1994). Moreover, evidence suggests that only canonical NF-κB signaling is oxygen-sensitive (Oliver et al., 2009; Taylor and Cummins, 2009). Interestingly, both HIF-1α and IKKβ were found to possess a highly conserved LxxLAP motif (Figure 4; Supplementary Table S1), which contains sites for prolyl hydroxylation. Research has shown that PHD-2 activity is necessary for the downregulation of NF-κB activity (Cummins et al., 2006; Takeda et al., 2011). Therefore, PHD-2 is a vital regulator of the oxygen sensing mechanism because it regulates the activity of both transcriptional factors important in regulating cellular responses to hypoxia. It is pertinent to mention that FIH-1 also hydroxylate various members of the NF-κB pathway, such as p105 and IκBα, but its significance on NF-κB remains elusive (Cockman et al., 2006).

### 3.3 Biological effects of HIF-1α in tumor cell

#### 3.3.1 HIF-1α and glucose metabolism

HIF-1α was initially recognized as a transcriptional activator of erythropoietin gene (EPO), was found to upregulate the expression of genes encoding glycolytic enzymes and glycolytic flux (GLUT1 and GLUT3), indicating that HIF-1α is an essential factor contributing to the “Warburg effect” (Samanta and Semenza, 2018).

First, it seems that OXOPHOS inhibition results due to a lack of oxygen, but this is not the case. HIF-1α achieves maximum stability at 1% O_2_ due to inactivation of FIH, but OXOPHOS can occur at even lower O_2_ concentrations (0.1%–0.7%) (Kennedy and Jones, 1986; Wilson et al., 1998; Stolze et al., 2004). This provides sufficient evidence that HIF-1α is stable well before O_2_ becomes limiting for OXPHOS. Therefore, HIF-1α has an instrumental role in the transfer of energy production from OXOPHOS to aerobic glycolysis.

**FIGURE 5:**
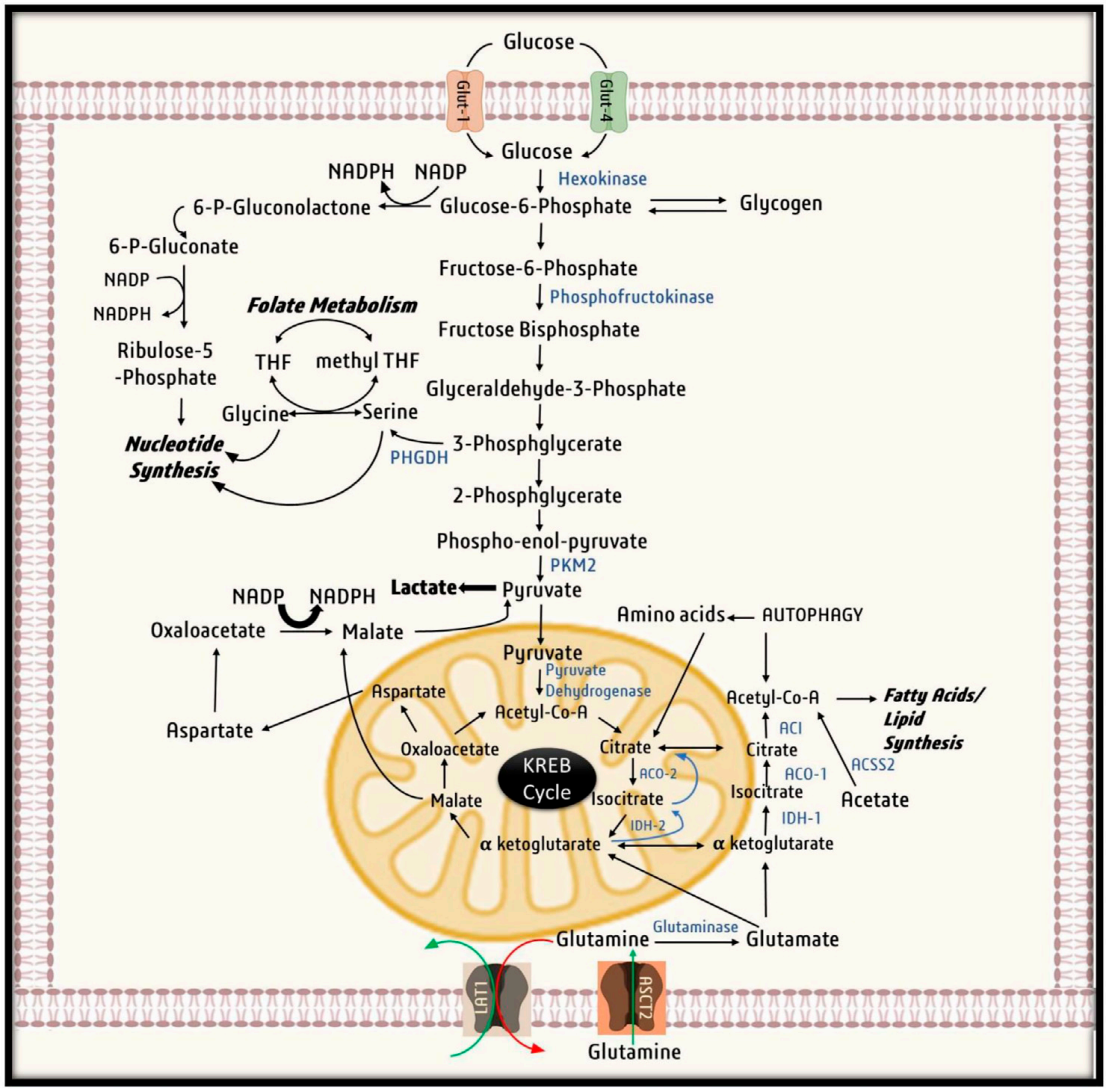
Utilization of Glucose by cancer cells. Cancer cell uptake Glucose inside the cell with the help of glucose transporters (GLUTs). Glucose then undergoes glycolysis, in first step of glycolysis, glucose is converted to Glucose-6-phosphate by Hexokinase, that is utilized by cancer cells for nucleotide synthesis and production of NADPH by PPP shunt. When in abundance glucose-6-phosphate is stored in form of glycogen. Glucose -6-phosphate then gets converted to 3-phosphoglycerate to provide for nucleotide synthesis through folate metabolism. Ultimately in the last step of glycolysis, phospho-enol-pyruvate is converted to pyruvate. Due to Warburg effect pyruvate is converted to lactate and excreted out of the cell. Under mild hypoxic conditions, pyruvate derived from malate aspartate shuttle and exogenous lactate as well as oxidative metabolism of glutamine runs the Krebs cycle to generate ATP. On the other hand, under severe hypoxia due to activation pyruvate kinase-1, pyruvate does not undergo conversion to citrate instead utilized for other vital process of cell survival. While citrate is aerobically generated from glutamine by conversion of glutamine to glutamate and ultimately to α-ketoglutarate. α-ketoglutarate is further converted by Isocitrate dehydrogenase-2 to isocitrate and then citrate by acoitase-2. Ultimately, citrate is converted to acetylcoA for fatty acid synthesis.

It is well established that HIF-1α is the key to the production of nucleotides, amino acids, fatty acids, lipids, and glycogen for the synthesis of cellular components in hypoxic microenvironments. In such cases, glucose is required to maintain cell energetics and provide biosynthetic intermediates such as ribose-5-phosphate, one carbon for nucleotide synthesis, and amino acids for protein synthesis resulting in an upsurge in demand for glucose. To counter such glucose crises, HIF-1α adapts to several mechanisms (Figure 5). Research has shown that malignant cells can compensate for the loss of glucose by utilizing intracellular glycogen for their survival and proliferation. Moreover, cancer cells can increase their glycogen accumulation by inducing enzyme glycogen synthase, which serves as a glucose reserve for the pentose phosphate pathway (Pescador et al., 2010). It has also been shown that HIF-1α upregulates the expression of glycogen phosphorylase, an enzyme required for glycogen metabolism. Research indicates that cancer cells target glycogen metabolism *via* the liver by glycogen phosphorylase to escape p53 dependent senescence *via* repression of ROS generation (Favaro et al., 2012).

Recent research has shown that some cancer cells, such as glioblastoma and breast cancer, utilize exogenous acetate to acetyl-CoA for lipid synthesis and biomass accumulation (Mashimo et al., 2014; Schug et al., 2015). The enzyme catalyzing this reaction is acetyl CoA synthase −2, an important target gene of HIF-1α and is integral to cancer cell survival and proliferation under hypoxic conditions (Kamphorst et al., 2014). These findings suggest that to meet the energy requirement of rapidly proliferating cells, HIF-1α shifts metabolism from oxidative phosphorylation to aerobic glycolysis. To manage glucose crises, HIF-1α facilitates glycogen and acetate by activating enzyme glycogen synthase and glycogen phosphorylase in hypoxic cancer cells.

#### 3.3.2 HIF-1α regulator of cellular acidity and redox homeostasis

HIF-1α activates pyruvate dehydrogenase kinase-1(PDK1), which phosphorylates and renders mitochondrial pyruvate dehydrogenase ineffective. As a result, glycolytic pyruvate is not converted to acetyl-CoA and NADH, decreasing the NADH flux to the electron transport chain (ETC) and production of ROS. The pyruvate thus accumulated undergoes conversion to lactate by the activity of lactate dehydrogenase A (LDHA) (Semenza et al., 1996; Papandreou et al., 2005). The lactate generated increases the cytosolic acidity and inhibits glycolysis. Therefore, lactate is effluxed out of the cell through a cell surface transporter known as MCT-4, which ultimately contributes to extracellular acidification and provides a favorable microenvironment for cellular proliferation. Henceforth, HIF-1α, through direct regulation of PDK1, LDHA, and MCT-4, effectively saves cancer cells from acidosis and harmful reactive oxygen species (Gatenby et al., 2007; Pinheiro et al., 2012; Doherty and Cleveland, 2013; Singh et al., 2023). Past research suggests that HIF-1α dependent expression of PDK-1 is required for metastatic colonization of breast cancer cells in the liver, contrary to lung or bone metastasis (Dupuy et al., 2015). Finally, to combat toxic ROS, HIF-1α indirectly induced the expression of glutathione. Since glutathione synthesis requires NADPH, HIF-1α triggers HMP shunt by initiating O-GlcNAcylation of glucose 6-phosphate dehydrogenase (Rao et al., 2015). Therefore, to protect cancer cells from harmful ROS, HIF-1α activates PDK-1, impairs the Kreb cycle, thereby reducing NADH flux to the electron transport chain and generating ROS. Moreover, HIF-1α also increases NADPH production from the HMP pathway to upregulate glutathione synthesis to maintain redox homeostasis.

#### 3.3.3 Modulation of lipid and fatty acid synthesis

Fatty acid synthesis requires the activity of ATP citrate lyase for the conversion of Kreb-cycle-derived citrate into acetyl-CoA. Acetyl-CoA undergoes carboxylation in the presence of the enzyme acetyl-CoA carboxylase (ACC) to produce malonyl-CoA, which is subsequently grouped into long-chain fatty acids by the enzyme fatty acid synthase (FASN) (Pavlova and Thompson, 2016).

Many studies have reported that FASN activity is required for lipid biosynthesis and cellular proliferation in various cancer pathologies (Currie et al., 2013; Zaidi et al., 2013). Moreover, downregulation of FASN was found to promote breast cancer prognosis (Roy et al., 2020; Singh et al., 2021b; 2021a) (Singh et al., 2016; Roy et al., 2017; Singh M. et al., 2018; Devi et al., 2019a). It has been suggested that FASN activity is upregulated in response to an increase in the expression of SREBP-1c by HIF-1α (Furuta et al., 2008; Singh L. et al., 2018). Fatty acid, triacylglycerol, and cholesterol synthesis under hypoxia are supplemented by fatty acid uptake from the extracellular compartment by upregulating PPARγ and fatty acid-binding protein (FABP-3,7) whose transcription is regulated by HIF-1α (Krishnan et al., 2009; Bensaad et al., 2014). Furthermore, HIF-1α enhances the endocytosis of lipoproteins by increasing the number of cell surface receptors, LRP-1 (lipoprotein receptor −1) and VLDL receptor (VLDLR) (Perman et al., 2011).

In addition, fatty acids produced by overexpression of FASN are utilized for membrane synthesis by conversion into phospholipids or stored in the form of triacylglycerols in lipid droplets. Moreover, the esterification of free fatty acids to neutral TAGs and their storage in lipid droplets saves cells from lipotoxicity (Mukerjee et al., 2021). It prevents cancer cells from intratumoral hypoxia from harmful free radicals generated during the cycles of hypoxia and reoxygenation (Young et al., 2013; Bensaad et al., 2014; Yoo et al., 2014; Ackerman et al., 2018). During harsh times, cancer cells can utilize lipid droplets to produce signaling molecules, such as sphingosine 1, as well as for ATP production *via* β-oxidation in mitochondria (Ackerman and Simon, 2014). Previous findings suggest that hypoxia induces HIF-1α dependent lipid droplet accumulation in tumor cells (Mylonis et al., 2012; Kourti et al., 2015). Moreover, phosphorylation of HIF-1α can prevent lipid droplet accumulation and malignant cell proliferation under hypoxic (Mylonis et al., 2012; Karagiota et al., 2019). Henceforth, HIF-1α fulfills the fatty acid requirement of rapidly proliferating cells by inducing expression of FASN, PPARγ, and FABP-3.7 which increases the production of fatty acid and ensures their storage in the form of lipid droplets. These lipid droplets act as a reservoir of fatty acid during times of starvation and protect cells from lipotoxicity and harmful ROS.

#### 3.3.4 HIF-1α mediated angiogenesis, metastasis and invasion

Under hypoxic conditions, HIF-1α regulates a vast set of genes and pro-angiogenic factors both in tumor mass and vascular endothelial cells, including vascular endothelial growth factor (VEGF) (Forsythe et al., 1996), angiopoietin-2 (ANGPT-2) (Simon et al., 2008), stromal-derived factor-1α (SDF-1α) (Ceradini et al., 2004), stem cell factor (SCF) (Bosch-Marce et al., 2007), and platelet-derived growth factor-β (PDGF-β) (Kelly et al., 2003). All of these are crucial players in the sequence of events involved in tumor angiogenesis. VEGF is pivotal not only for endothelial cell activation and proliferation through VEGF-R signaling but is also responsible for reduced vascular endothelial cell apoptosis by the upregulation of BCL-2 (Gerber et al., 1998). VEGF is also secreted in a paracrine fashion from pericytes in response to PDGF secreted from tumor cells (Reinmuth et al., 2001). HIF-1α also governs vascular tone by modulating NOS (Rey and Semenza, 2010). ANGPT-2 weakens the interactions between endothelial cells and smooth muscle cells, responsible for endothelial cell migration (Maisonpierre et al., 1997). *In vivo* investigations have reported that targeting HIF-1α remarkably inhibits tumor vascularization (Lee et al., 2009). HIF-1α induces metastasis, which is the primary cause of tumor-related deaths (Balamurugan, 2016). Limitations of tumor metastasis and invasiveness rests upon HIF-1α mediated transcriptional activation of matrix metalloproteinases (MMPs) and enzyme lysyl oxidase (Wong et al., 2011). Reports reveal that HIF-1α directly promotes epithelial to mesenchymal transition (EMT) by inducing the loss of E-cadherin (Krishnamachary et al., 2006; Zhang et al., 2014). It has been found that HIF-1α, through direct regulation of the TWIST gene, induces metastasis (Yang et al., 2008). CXCR-4 and urokinase-type plasminogen activator receptor (uPAR) are important receptor targets of HIF-1α in hypoxia-mediated metastasis (Staller et al., 2003; Büchler et al., 2009). The above studies suggest that the cells which are at the center of tumor mass require blood supply for transport of O_2_, nutrients, and growth factors; therefore, HIF-1α activates angiogenic factors such as VEGF, ANGPT-2, SDF-1α, SCF, and PDGF-β to facilitate neoangiogenesis, endothelial cell proliferation, and endothelial cell migration. Moreover, HIF-1α also induces tumor dissemination to distal cites by directly activating TWIST, CXCR-4, and uPAR.

#### 3.3.5 HIF-1α and apoptosis

Hypoxia plays a dual role in the regulation of apoptosis under graded oxygen conditions. A study reported that cells treated with staurosporine were less sensitive to apoptosis under severe hypoxia (0.1% oxygen) (Dong et al., 2003). HIF-1α has both pro-and anti-apoptotic effects. Pro-apoptotic activity occurs from continuous or extreme hypoxia, which arises from the interaction of HIF-1α with p53. The anti-apoptotic activity of-HIF-1α arises at the level of O_2_ at which HIF-1α can dimerize with ARNT, thereby increasing the transcription of anti-apoptotic genes (Piret et al., 2002).

Another study reported that two different forms of HIF-1α should be held liable for various activities. The phosphorylated form of HIF-1α dimerizes with ARNT and triggers antiapoptotic genes. On the other hand, dephosphorylated HIF-1α interacts with p53, thereby stabilizing p53, which induces apoptosis *via* BAX overexpression (Suzuki et al., 2001). Apart from BAX, BNIP3 and NIX are also overexpressed at the transcriptional level during hypoxia. When oxygen is sparse, HIF-1α promotes mitophagy and inhibits mitochondrial biogenesis by upregulating BNIP3 expression and inhibiting c-Myc-dependent mitochondrial generation (Sowter et al., 2001; Zhang et al., 2007; Zhang et al., 2008). The above studies suggest that below 0.1% oxygen (wherein both PHD-2 and FIH are inactive), HIF-1α can dimerize with ARNT and activate transcription of genes that inhibit apoptosis. Whereas, pro-apoptotic effects of HIF-1α may be attributed to its cross-talk p53 which need further investigation.

## 4 Bioenergetics of normal cells *versus* cancer cells

The energy requirement of the cell is fulfilled by the catabolism of carbohydrates, proteins, and fat. The energy production starts with the catabolism of carbohydrates to glucose, which is further converted to pyruvate *via* a set of ten enzymatic reactions forming a pathway known as “Glycolysis” (DeBerardinis et al., 2008). In brief, glycolysis is a two phase process. First, is the “investment phase” in which ATP is utilized to produce high energy intermediates (such as conversion of glucose to glucose-6- phosphate by hexokinase and conversion of fructose -6-phosphate to fructose-1,6-bisphosphate by phosphofructokinase). Second is the “pay off phase” in which high energy intermediates are broken down to generate ATP molecules (such as conversion of 1,3-bis phosphoglycerate to 3-phosphoglycerate by phosphoglycerate kinase and conversion of phosphoenolpyruvate to pyruvate by pyruvate kinase). Furthermore, glycolysis also generates NADH from conversion of glyceraldehyde-3-phosphate to 1,3-bis phosphoglycerate by glyceraldehyde-3-phosphate dehydrogenase (GAPDH) (Chandel, 2015).

Overall, glycolysis contributes to the generation of four ATPs and two NADH molecules from one molecule of glucose. However, the utilization of two ATPs in the investment phase reduces the net gain to 2-ATPs and 2 NADH. Therefore, if we spare NADH, which requires oxygen for energy production, at least two ATPs are generated from substrate-level phosphorylation whenever a glucose molecule undergoes glycolysis (Bonora et al., 2012). The end product of glycolysis, pyruvate, yields different products under aerobic and aerobic conditions. In the absence of oxygen, pyruvate is transformed to lactic acid by lactate dehydrogenase A (LDHA) and is expelled out of the cell (Semenza et al., 1996).

In contrast, in the presence of oxygen, pyruvate is transformed to acetyl-CoA under the catalytic influence of the pyruvate dehydrogenase enzyme. This acetyl-CoA yielded from pyruvate (from glycolysis) combined with oxaloacetate to generate citrate. This marks the start of energy production, which is inside the mitochondria called the “Citric acid cycle.” The citric acid cycle liberates essential metabolic intermediates, such as alpha-ketoglutarate, succinate, fumarate, malonate, and oxaloacetate. These intermediates are metabolic precursors of amino acids, nucleotides, fatty acids, hemes, and porphyrins, which are essential components for synthesizing cell membranes, DNA, proteins, and other macromolecules (DeBerardinis et al., 2008).

It is appropriate to mention that similar to glycolysis, the TCA cycle can generate two ATPs from substrate-level phosphorylation, which occurs when the succinic CoA synthetase enzyme catalyzes the reversible reaction of succinyl CoA to succinate (Galluzzi et al., 2010). The oxidation of acetyl-CoA to CO2 by the electron transport chain reduces NAD+ and FAD+ to form high-energy carriers NADH and FADH2 during the cycle. For this reason, the Kreb cycle, though it does not require oxygen for functioning, requires oxygen for the oxidation of NADH and FADH2 to yield NAD+ and FAD+ by an electron transport chain (or oxidative phosphorylation), which is coupled to the Kreb cycle (Martínez-Reyes and Chandel, 2020). In normal cells, ATP is generated mainly from OXOPHOS, accounting for nearly 89% of the total cellular energy, while substrate-level phosphorylation contributes 11% of the whole cellular energy. Approximately 32–38 total ATP molecules are generated from the complete oxidation of one molecule of glucose. Cancer cells switch from oxidative phosphorylation to glycolysis for most of their energy requirements. This prevents the generation of harmful reactive oxygen species (ROS) by ETC (ROS are the product of electron acceptation by O_2_ in ETC) and facilitates rapid ATP supply to fast proliferating cells since glycolysis is a quicker process than oxidative phosphorylation. This dependency of cancer cells on glycolysis and excessive production of lactate from pyruvate, despite the presence of O_2_, is termed the Warburg effect (Warburg, 1925; Epstein et al., 2014; Shestov et al., 2014; Warburg Berlin-Dahlem, 2016).

## 4 Energy generation and transcriptional regulation of tumor cell proliferation under graded oxygen tension: Role of lactate shunt

**FIGURE 6:**
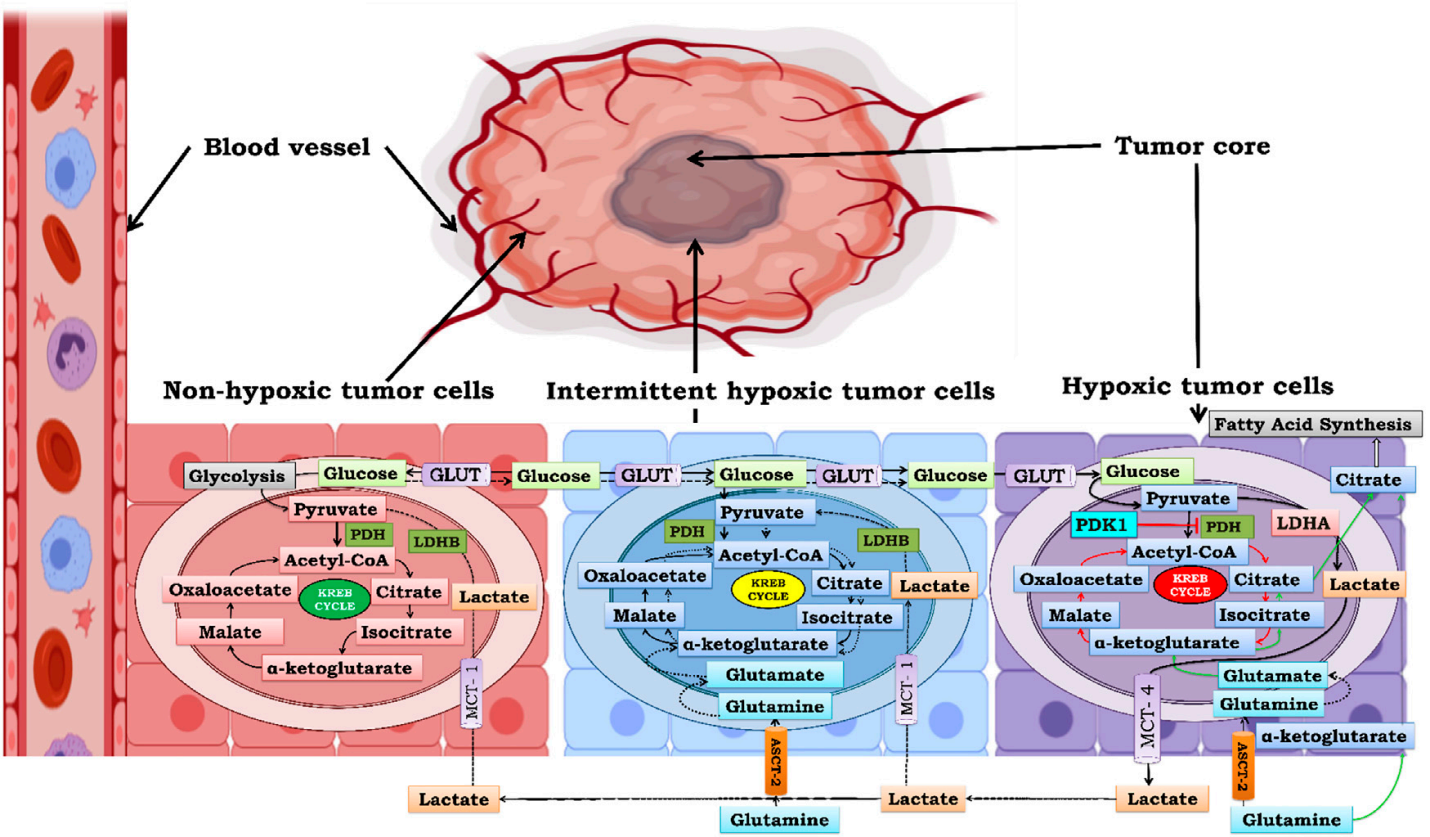
Metabolic Symbiosis under graded oxygen to produce ATP. Bioenergetics involved in solid tumor is highly complex. Cells which are closed to blood vessels have normal supply of oxygen and glucose therefore obtain energy through glycolysis and kreb cycle initially but as the size of the tumor increases the demand of glucose increases more and more to compensate for this ever increasing demand of glucose there is an upsurge in MCT-1 expression in non-hypoxic cells and they take up exogenous lactate from extracellular tumor microenvironment and convert it to pyruvate by the action of enzyme lactate dehydrogenase B (LDHB). and further this extemporaneously generated pyruvate is utilized to gain energy through Kreb cycle thus sparing glucose for utilization by hypoxic areas which are far from blood vessels. Second in line are intermittently hypoxic cells which utilize both exogenous lactate as well as oxidative glutamine metabolism for energy requirement. Ultimately the core of tumor is formed by severely hypoxic cells which lack access to both blood and oxygen therefore glycolysis is the only source for energy replenishment and therefore undergo rapid glycolysis and generate huge amount of lactate resulting in lactic acidosis. Therefore, in order to survive cancer cells, excrete this lactate out of the cell. This excreted lactate serves as pool of extracellular lactate which can be taken by non-hypoxic and intermittent hypoxic cells for energy production. Hence this cycle goes on and on leading to increase in tumor mass by rapid cellular proliferation and survival.

As previously discussed, as tumors grow away from blood vessels, areas that are distal to blood vessels or sometimes due to poor vasculature experience graded oxygen tension (Campillo et al., 2019). Various models have been proposed previously, which classify the tumor into two zones: the non-hypoxic zone, which has an adequate supply of oxygen to support tumor cell growth, and the second is the hypoxic zone, which lacks oxygen. In solid tumors, it is important to consider a third zone that is intermittent hypoxic simply because tumors have intermittently hypoxic patches. Therefore, to understand how cells of the same tumor respond differently to different pO_2_, we bifurcated the tumor cells into three zones based on the oxygen gradient (Figure 6). The three zones are i) non-hypoxic zone (40 < pO_2_ < 160 mmHg), ii) intermittent hypoxic zone (10 < pO_2_ < 40 mmHg), and iii) severely hypoxic zone (pO_2_ < 10 mmHg) (McKeown, 2014). To survive, cells in these zones cooperate with each other to fulfill their metabolic needs called “Metabolic Symbiosis.” In recent times, lactate has evolved as the molecule which facilitates this “Metabolic Symbiosis” (Guppy et al., 1993). In the subsequent section, we will see how lactate facilitates cells to tackle energy crises in all three zones.

The non-hypoxic zone (40 < pO_2_ < 160 mmHg) comprised oxygenated tumor cells. These cells have free access to O_2_ because they lie close to the blood vessels. In the early phase of tumor development, these cells prefer glycolysis over oxidative phosphorylation because glycolysis can supply ATP faster to rapidly proliferating cells as compared to oxidative phosphorylation and also reduces the oxidative stress on tumor cells (Guppy et al., 1993; Pfeiffer et al., 2001). However, as the size of the tumor increases, a large amount of glucose is required not only to maintain cell energetics but also to provide biosynthetic intermediates such as ribose-5-phosphate, one carbon for nucleotide synthesis, as well as amino acids for protein synthesis, resulting in an increase in the demand for glucose. Therefore, oxygenated tumor cells reduce glucose uptake and increase lactate uptake from the extracellular environment during tumor development. Lactate is picked up from the extracellular environment through MCT-1 and is converted to pyruvate by LDHB. Pyruvate generated from lactate enters the Krebs cycle and provides energy through oxidative phosphorylation. This metabolic switch from glycolysis to oxidative phosphorylation provides a large number of ATPs and TCA cycle intermediates and makes glucose readily available for the survival of hypoxic cells that are distal to blood supply (Feron, 2009).

The intermittent hypoxic zone (10 < pO_2_ < 40 mmHg) is the connecting link between the non-hypoxic zone and severely hypoxic zone, and therefore acts as a bridge that maintains a continuous supply of glucose from the non-hypoxic location the periphery to the severely hypoxic zone at the center. Similar to non-hypoxic cells, these cells rely mainly on the Kreb cycle and oxidative phosphorylation to generate ATP by utilizing pyruvate generated from lactate, thereby sparing glucose for utilization in severely hypoxic zones (Sonveaux et al., 2008; Nakajima and Van Houten, 2013). Therefore, these cells are also known as OXOPHOS cells. In addition to exogenous lactate uptake, OXOPHOS cells also take up glutamine for oxidative glutamine metabolism. In oxidative glutamine metabolism, glutamine is converted to glutamate by glutaminase, and glutamate dehydrogenase converts glutamate to the TCA cycle intermediate 2-oxoglutarate. Anaplerotically, 2-oxoglutarate undergoes oxidative glutamine metabolism, producing energy and rejuvenating the pool of Kreb cycle intermediate precursors of nucleotides, amino acids, and lipids (Moreadiths and Lehningert, 1984; DeBerardinis et al., 2007).

The severely hypoxic zone (pO_2_ < 10 mmHg) consists of glycolytic cells that obtain energy from glycolysis only due to impairment of mitochondrial oxidative phosphorylation. Citrate deficit arising from the impaired mitochondrial function is partially fulfilled by “anaplerosis” from reductive glutamine metabolism in these cells (Moreadiths and Lehningert, 1984; DeBerardinis et al., 2007). Impairment of oxidative phosphorylation by mitochondrial defects is attributed to the activation of HIF-1α, which causes selective mitophagy to reduce oxygen consumption and ROS production under hypoxia (Zhang et al., 2008). Moreover, HIF-1α activates the enzyme pyruvate dehydrogenase kinase-1 (PDK1), which phosphorylates and thereby blocks the function of mitochondrial pyruvate dehydrogenase, which converts pyruvate to acetyl-CoA, thereby uncoupling pyruvate from the Kreb cycle [114]. HIF-1α further induces the enzyme LDHA, which oxidizes pyruvate to lactate, and in the process, NADH is oxidized to NAD+ (Semenza et al., 1996). NAD + functions as an electron acceptor in the glycolytic pathway and therefore plays a vital role in the continuous running of glycolysis to support the energy requirements of rapidly proliferating cells (Bui and Thompson, 2006; Fan et al., 2011). Lactate, a by-product of glycolysis, is effluxed out of the cell by monocarboxylate transporter-4 (MCT-4), which is also a transcriptional target of HIF-1α (Pinheiro et al., 2012). Extracellular lactate accumulation favors migration and metastasis and acts as a reservoir of energy for non-hypoxic and OXOPHOS cells (Goetze et al., 2011; Dhup et al., 2012; Brooks, 2018).

From the above, it is clear that “metabolic symbiosis between the three zones is necessary for holistic growth and proliferation of cancer cells, and lactate shunt is the key to this metabolic adaptation.” Compared to energy production, tumor growth and proliferation regulation under graded oxygen tension are much more complex. Studies over the past decade have emphasized HIF-1α as the central regulator of tumor cell survival, proliferation, angiogenesis, and metastasis under graded oxygen tension (Schofield and Ratcliffe, 2004). Here, we focus on the intricacies tangled in the modulation of HIF-1α in the non-hypoxic, intermittent hypoxic, and severely hypoxic zones.

In the non-hypoxic zone (40 < pO_2_ < 160 mmHg), both PHD-2 and FIH-1 remain functional, which contribute to proteasomal degradation and transcriptional inactivation of HIF-1α, respectively (Maxwell et al., 1999; Bruick, 2001; Lando et al., 2002; Kaelin, 2003). In intermittent hypoxic zones (10 < pO2 < 40 mmHg), PHD-2 is inactivated while FIH-1 remains functional (Koivunen et al., 2004; Stolze et al., 2004). Therefore, how HIF-1α regulates the transcriptional factors responsible for cell growth and proliferation in these zones remains a question. Research postulates that Lactate uptake from the extracellular environment and its subsequent conversion to pyruvate by LDHB enzyme could be responsible for non-hypoxic activation of HIF-1α since pyruvate acts as a competitive inhibitor of oxoglutarate (α-ketoglutarate), which is a co-substrate for enzymes PHD-2 and FIH-1. Therefore, reduced levels of α-ketoglutarate might lead to the inactivation of PHD-2 and FIH-1, thereby facilitating the stabilization and transcriptional activity of HIF-1α (De Saedeleer et al., 2012). Notably, it has been found the Michaelis constant (Km) of FIH for the substrate α-ketoglutarate is 55–60 µM, which is just double that of PHD-2, whose Km value for α-ketoglutarate is 25 µM (Koivunen et al., 2004).

Moreover, it has been found that FIH remains functional up to pO_2_ = 10 mmHg, while PHD-2 is inactivated as soon as pO_2_ reaches <40 mmHg. Therefore, even if PHD-2 is inactivated by competitive inhibition of α-ketoglutarate by pyruvate, FIH-1 remains active and prevents HIF-1α transcriptional activity (Rani et al., 2022). Thus, pyruvate-induced competitive inhibition of α-ketoglutarate will only contribute to the stability of HIF-1α but not to the functional activity of HIF-1 α in non-hypoxic cells. Now, a question still remains: how does tumor cell survival, proliferation, angiogenesis, and metastasis occur in the truancy of HIF-1α. This raises an intriguing possibility that other transcriptional regulators are involved that facilitate cellular adaptation when oxygen tension is limiting for HIF- 1α.

In severely hypoxic zones (pO_2_ < 10 mmHg), both PHD-2 and FIH-1 are inactivated in response to oxygen. As a result, HIF-1α becomes stable and activates genes responsible for tumor cell growth survival.

## 5 Different hypotheses for NF-κB activation and their future endorsement: Role of PHDs

### 5.1 PHDs as a dual regulator of HIF-1α and NF-κB

While HIF-1α is an extensively studied transcriptional regulator under hypoxia, other transcriptional regulators exist (Nakayama and Kataoka, 2019). NF-κB is also a transcriptional regulator. NF-κB is a crucial modulator of angiogenesis, metastasis, migration, cell survival, and proliferation (Gilmore, 2006), but its role in conjunction with HIF-1α has not been studied extensively. A few studies have established a link between HIF-1α and NF-κB; however, the results have been contradictory. Although it appears that both HIF-1α and NF-κB work hand in hand, establishing a well-defined mechanism has proven elusive (BelAiba et al., 2007; Nam et al., 2011; Azoitei et al., 2016).

Interestingly, PHDs that regulate HIF-1α activity were also found to regulate NF-κB signaling through IKKβ. An *in vitro* study showed that inhibition of PHDs moderately activated IKKβ in cells cultured under hypoxic conditions (Walmsley et al., 2005). It would be appropriate to mention that NF-κB activation is regulated by IKKβ induced phosphorylation and degradation of IκB inhibitors (IκBα and IκBβ). It has been reported that ischemia leads to hypoxia, which can suppress PHD1 function, thereby enhancing IKKΒ expression for NF-κB activation (Cummins et al., 2006; Xie et al., 2014).

It was also found that PHD-2 downregulation increased NF-κB-mediated expression of IL-8 and angiogenin in certain tumors. The same study also reported that stimulation of NF-κB activity by TNF-α treatment was impaired in the presence of PHD-2, which further confirms that PHD-2 is a negative modulator of NF-κB (Chan et al., 2009). Moreover, a functional link between both ROS and PHD2 pathways was provided by showing that the conversion of NAD + to NADH and H+ by LDHB was converted from lactate to pyruvate.

Competitively inhibits PHD. but also stimulates NAD(P) H oxidase (Végran et al., 2011). NAD(P) H oxidase generates ROS from a pool of NADH. ROS generated may contribute to the inhibition of PHD-2, leading to an increase in basal NF-κB activity (Gerald et al., 2004; Pan et al., 2007). From the above findings, there appears to be a negative regulatory loop between PHDs and NF-κB.

### 5.2 Various mechanisms of NF-κB activation

Endothelial cells have an increased expression of MCT-1, which allows them to take up a large amount of lactate from the extracellular environment. Although MCT-1 operates bi-directionally depending on the concentration gradient, the unidirectional movement of lactate is maintained by its higher affinity for lactate (Km = 3–6 mM) and an ever-increasing extracellular pool of lactate effluxed from glycolytic cells through MCT-4 (Km = 25–30 mM) (Halestrap, 2013; Benjamin et al., 2018).

**FIGURE 7:**
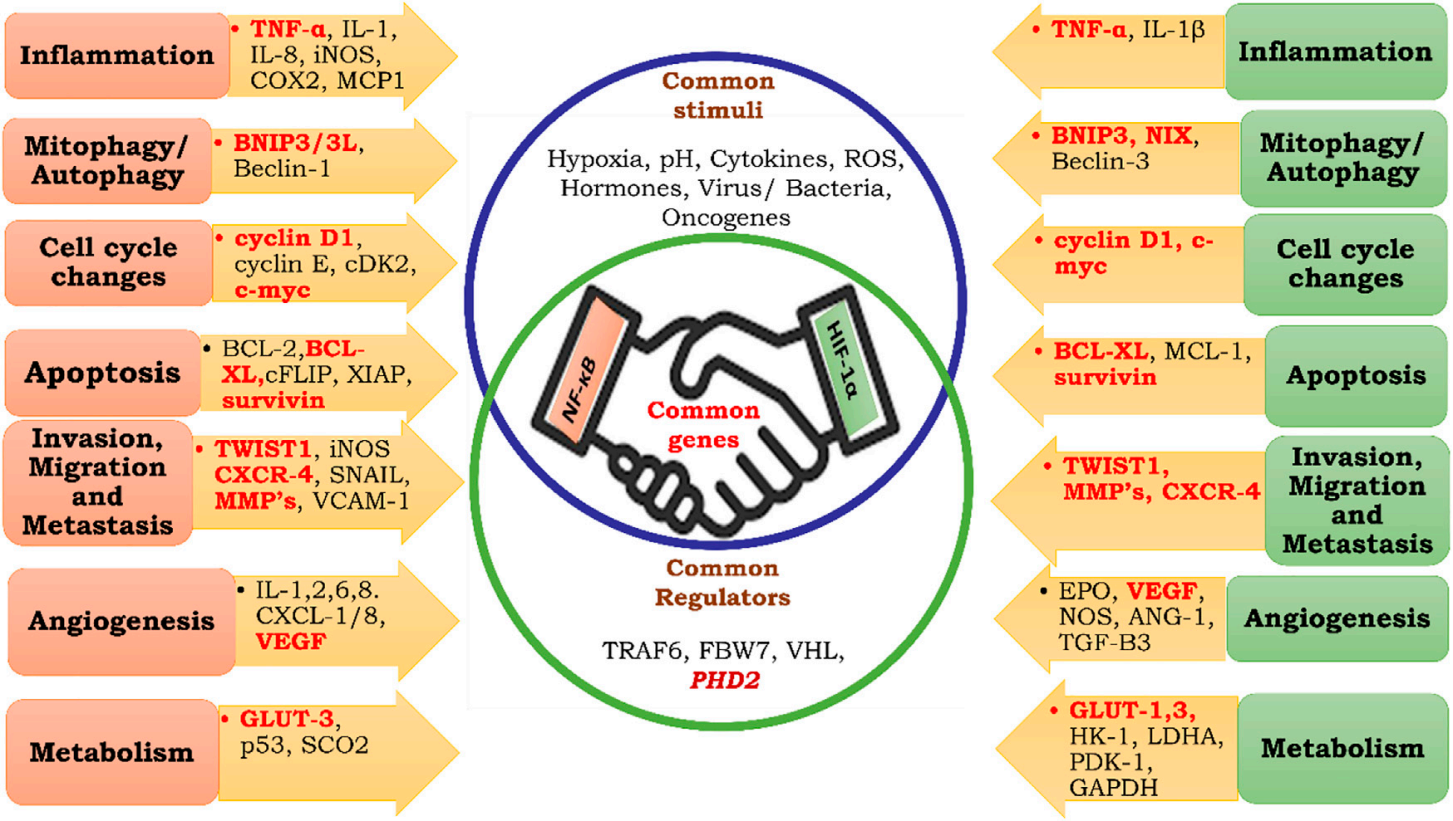
Common targets of HIF-1α and NF-κB. Inflammation, Mitophagy, Autophagy, Cell Cycle Changes, Apoptosis, Invasion, Migration, Metastasis, Angiogenesis, Metabolism.

This effluxed lactate is converted to pyruvate in the presence of the enzyme LDHB to run the Krebs cycle for energy production, thereby sparing glucose for distal hypoxic areas [171] (Figure 7). However, LDHB converts NAD+ to NADH and H+, thus generating NAD(P) H oxidases. NAD(P) H oxidases produce large amounts of ROS. Moreover, pyruvate, which is a competitive inhibitor of 2-oxoglutarate, inhibits PHD2. Both excessive ROS production and inhibition of PHD2 contribute to NF-κB activation (Gatenby and Gillies, 2004). It is pertinent to mention that MCT-4 plays a pivotal role in NF-κB activation indirectly by effluxing lactate out of the cell, which is further taken up by MCT-1 from the extracellular environment.

Specific blocking of MCT-1 could be a possible mechanism to inhibit NF-κB activity by preventing competitive inhibition of PHD-2 and generation of ROS. On the other hand, specific blockage of MCT-4 leads to decreased extracellular lactate levels, creating a negative feedback loop, thereby inhibiting lactate uptake by MCT-1. Blocking both MCT-1 and 4 could be a more dynamic approach because it would disrupt the metabolic symbiosis of lactate both intracellularly and extracellularly, rendering NF-κB ineffective.

Activation of NF-κB by this mechanism is dependent on the uptake of lactate; however, there is a need to investigate the level at which cells would prefer to take lactate instead of glucose when both are available in abundance. Secondly, cells could hire other mechanisms to overcome the effect of blockage of MCTs and increase intra-or extracellular acidity.

Modern research has focused on inhibiting the enzyme LDHB, a crucial enzyme involved in converting lactate to pyruvate. Inhibition of the activity of this enzyme has resulted in decreased disease prognosis in breast, colon, and ovarian tumors. This enzyme not only replenishes NADPH for fatty acid synthesis but also maintains redox homeostasis. This enzyme indirectly activates HIF-1α because of the competitive inhibition of PHD2 by pyruvate. However, whether competitive inhibition of pyruvate leads to activation of NF-κB remains a matter of research (Brisson et al., 2016; Mishra and Banerjee, 2019). Few researchers have suggested that competitive inhibition of PHD2 does not activate NF-κB in oxidative tumor cells but activates HIF-1α (De Saedeleer et al., 2012; Van Hée et al., 2015). It has been shown that PHD2 activation results in decreased activation of HIF-1α and NF-κB; thus, how the inhibition of the same regulating factor results in reduced expression of HIF-1α and not of NF-κB requires further investigation.

## 6 Unresolved riddle and proposed hypothesis.

It has been known for quite some time that inflammation, when left unchecked, might eventually result in cancer. The precise mechanism driving this event, however, remains unclear. Many studies (Höckel and Vaupel, 2004) have pointed to hypoxia as a prognostic factor that changes normal cells into cancerous ones in a chronic inflammatory milieu. Previous studies have implicated NF-B and HIF-1α as two significant transcriptional regulators in this transition. But how they interact is still a mystery. The two transcription factors control separate aspects of cell growth and division. While HIF-1α promotes malignant cell adaptability and proliferation, nuclear factor-kappa B (NF-kappa B) is essential for tumour initiation and survival (Dolcet et al., 2005; Masoud and Li, 2015). Many studies have looked into the method by which these two proteins cooperate, however the results have been inconsistent. While some research suggests that HIF-1α activates following NF-κB suppression, others show that the opposite is true. Still other research suggests that NF-κB regulates the HIF-1-mediated response. Overall, little is known about HIF-1’s involvement with its inflammatory companion NF-κB. Basal NF-κB activity is essential for HIF-1α activation, and this has been stressed by a small but vocal group of researchers in recent years, although there is not yet enough evidence to back up these claims. Moreover, it is pretty understandable that sufficient oxygen is available in the early phase of tumorigenesis because the affected area is close to the blood vessels. Therefore, HIF- 1α undergoes proteasomal degradation due to the presence of PHD2. Although non-hypoxic stabilization of HIF-1α and NF-κB is very high on the cards by inhibition of PHD2 by ROS and TCA cycle intermediates (Lee et al., 2016), the presence of FIH is likely to seize the transcriptional activity of HIF-1α, while NF-κB undergoes transcriptional activation. Therefore, in the initial phase of tumors, it could be hypothesized that cellular proliferation is HIF-1α independent and NF-κB-dependent. It is pertinent to mention that PHD2 inhibition is necessary for the transcriptional activity of both HIF-1α and NF-κB.

**FIGURE 8:**
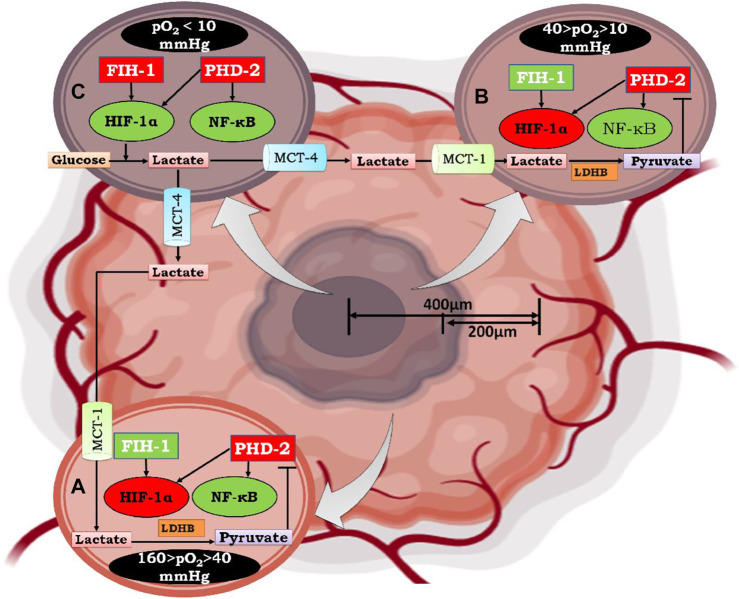
Mechanism of Lactate shuttle mediated activation of NF-κB under graded oxygen tension. As the tumor size increases to more than 200 μm, the diffusion of oxygen decreases, which marks the beginning of the condition known as “intratumoral” hypoxia. Moving from the periphery to the core (400 μm), cells are under graded oxygen tension, which ranges from 160 mmHg to less than 10 mmHg. Based on this, cells can be divided into three zones. In Figure A. represents a cell that lies in the non-hypoxic zone (160 > pO_2_ > 40 mmHg), B. represents a cell that lies in the intermittent hypoxic zone (40 > pO_2_ > 10 mmHg), and C. represents a cell that lies in a severely hypoxic zone (pO_2_ < 10 mmHg). In a non-hypoxic cancer cell (A) and intermittent hypoxic cancer cell (B), effluxed lactate enters through MCT-1 and is converted to pyruvate by the enzyme lactate dehydrogenase B. The conversion of lactate to pyruvate causes competitive inhibition of 2-oxoglutarate by pyruvate, which is the substrate for enzyme Prolylhydroxylase-2(PHD-2). Henceforth, PHD-2 fails to degrade IKKβ, resulting in the activation of NF-κB, which further regulates the downstream mechanism of cancer cell survival, growth, and proliferation. In contrast, HIF-1α, which assumes stability due to inactivation of PHD-2, does not undergo transcriptional activation because of hydroxylation at N803 of its C-TAD by factor inhibiting HIF-1α (FIH-1). Although FIH-1, similar to PHD-2, belongs to the group of 2-oxoglutarate dependent dioxygenases, has a Km value of 55-60μM for 2-oxoglutarate, which is double that of PHD-2, whose Km value for 2-oxoglutarate is 25 μM. Moreover, FIH-1 remained active up to an oxygen tension of 10 mm Hg. Therefore, FIH-1 effectively inhibits HIF-1α transcriptional activity in non-hypoxic and intermittent hypoxic zones. Henceforth, it can be postulated that in these zones cell survival and proliferation is under influence of NF-κB. In hypoxic cancer cells, lactate accumulates due to excessive glycolysis, which results in an increase in intracellular acidity. In order to cope with intracellular acidity, hypoxic cancer cells efflux lactate into the extracellular environment through monocarboxylate transporter-4 (MCT-4). Moreover, in hypoxic cancer cells, both PHD-2 and FIH-1 were inactive due to very low oxygen tension (pO_2_ < 10 mmHg). Thus, HIF-1α is both stable and transcriptionally active along with NF-κB. Therefore, cell survival and proliferation are influenced by both HIF-1α and the inflammatory partner NF-κB.

Past research suggests that NF-κB and HIF-1α share commonly targeted genes (Rocha, 2007; Park and Hong, 2016) (Figure 7); therefore, they both may be regulated by similar mechanisms. Despite the presence of FIH-1, angiogenesis, metastasis, migration, and other phenomena necessary for cellular proliferation take place. Therefore, it could be hypothesized that until oxygen becomes limiting for FIH, NF-κB regulates tumor cell growth and expansion through pyruvate-mediated competitive inhibition of PHD-2 (Figure 8).

## 7 Conclusion

To regulate HIF-1α and NF-κB, the PHD-2 is the typical checkpoint. Because of this, activating PHD-2 either directly or indirectly may represent a novel approach to treating solid tumours. PHD-2 may be directly activated by small-molecule chemical activators (Roy et al., 2018; Devi et al., 2019a; Roy et al., 2019). (Devi et al., 2019b). It is worth noting that there are just a few of compounds thought to be PHD-2 activators, and none of them have made it to market as of yet. In addition, the response in tandem with NF-κB downregulation is still not clear.

Activating PHD-2 in a roundabout way may be accomplished by focusing on MCT-1 and LDHB. Recent studies have indicated that inhibiting MCT-1 has a significant impact on cancer treatment, although their effect in tandem with PHD-2 activity has yet to be studied. As with Akt inhibition, LDHB inhibition was found to be useful in cancer therapy, but selective LDHB inhibitors have not been discovered as of yet. Consequently, it is important to study the impact of novel selective inhibitors of LDHB on carcinogenesis. The current study elucidates the function of NF-κB in the first stages of tumour development and proliferation, when HIF-1α is inactive due to elevated pO2. This review also sheds light on how, under normal oxygen tension, downregulation of PHD-2 by the lactate shuttle activates NF-κB but not HIF-1α. The review highlights PHD-2 as a dual down regulator of HIF-1α and IKK, and proposes that, like HIF-1α, PHD-2 causes hydroxylation and proteasomal degradation of IKK.

## Glossary

TNF-α: tumor necrosis factor-α
IL: interleukin
ROS: reactive oxygen species
PAMPs: pathogen associated molecular patterns
DAMPs: damage/danger associated molecular patterns
TLRs: toll like receptors
NF-κB: nuclear factor kappa light chain enhancer of activated B- cells
OXOPHOS: oxidative phosphorylation
MCT-1: monocarboxylate transporter-1
MCT-4: monocarboxylate transporter-4
LDHA: lactate dehydrogenase-A
LDHB: lactate dehydrogenase-B
GLUT-1: glucose transporter-1
GLUT-3: glucose transporter-4
HIF-1α: hypoxia inducible factor-1α
HIF-1β: hypoxia inducible factor-1β
PHD-2: prolylhydroxylase-2
ODDD: oxygen dependent degradation domain
pVHL: Von-hippel-Lindau tumor repressor protein
FIH-1: factor inhibiting hypoxia inducible factor 1α
CBP: CREB binding protein
CTAD: C- terminal transactivation domain
HRE: hypoxia response element
2-OG: 2- oxoglutarate
TCA: tricarboxylic Acid cycle
IκB: inhibitor of nuclear factor kappa B
IKK: inhibitor of nuclear factor kappa-B kinase
NEMO: NF-κB essential modulator
MEFs: mouse embryonic fibroblasts
TAK-1: transforming growth factor -β- activated kinase-1
SCF: SKP1-cullin-1-F- box protein
βTrCP: beta transducing repeats containing protein
VEGF: vascular endothelial growth factor
MMP’s: matrix metalloproteinases
CAF’s: cancer associated fibroblasts
ECM: extracellular matrix
CXCl-2: C-X-C motif chemokine ligand 2
SDF-1: stromal cell-derived factor*-*1
c-myc: cellular myelocytomatosis oncogene
CXCR4: C-X-C chemokine receptor type 4
bHLH: basic helix loop helix
PAS: PER-ARNT-SIM
ARNT: aryl hydrocarbon receptor nuclear translocator
ATP: adenosine triphosphate
ETC: electron transport chain
PDK-1: pyruvate dehydrogenase kinase-1
IDH-1: isocitrate dehydrogenase-1
IDH-2: isocitrate dehydrogenase-2
ACO-1/2: aconitase-1/2
ACl: ATP- citrate lyase
ACC: acetyl CoA carboxylase
FASN: fatty acid synthase
SREBP-1: sterol regulatory element binding protein-1
**PPAR**_**γ**_: peroxisome proliferator receptor-γ
FABP: fatty acid binding protein
LRP-1: lipoprotein receptor related protein
VLDLR: very low density lipoprotein receptor
TAGs: Triacylglycerols
AGPAT2: acylglycerol-3-phosphate acyl transferase-2
DAG: diacylglycerol
NHE1: sodium hydrogen exchanger-1
Ca9: carbonic anhydrase-9
BAFFR: B-cell activating factor receptor
CD: cluster of differentiation
RANK: receptor activator of nuclear factor κ B
TNFR2: tumor necrosis factor receptor 2
FN14: fibroblast growth factor-inducible 14
TWEAK: tumour necrosis factor (TNF)-like weak inducer of apoptosis

## References

[B1] Ackerman D. Simon M. C. (2014). Hypoxia, lipids, and cancer: Surviving the harsh tumor microenvironment. Trends Cell. Biol. 24, 472–478. 10.1016/j.tcb.2014.06.001 24985940 PMC4112153

[B2] Ackerman D. Tumanov S. Qiu B. Michalopoulou E. Spata M. Azzam A. (2018). Triglycerides promote lipid homeostasis during hypoxic stress by balancing fatty acid saturation. Cell. Rep. 24, 2596–2605. 10.1016/j.celrep.2018.08.015 30184495 PMC6137821

[B3] Akanji M. A. Rotimi D. Adeyemi O. S. (2019). Hypoxia-inducible factors as an alternative source of treatment strategy for cancer. Oxid. Med. Cell. Longev. 2019, 8547846. 10.1155/2019/8547846 31485300 PMC6710762

[B4] Azoitei N. Becher A. Steinestel K. Rouhi A. Diepold K. Genze F. (2016). PKM2 promotes tumor angiogenesis by regulating HIF-1α through NF-κB activation. Mol. Cancer 15, 3–15. 10.1186/s12943-015-0490-2 26739387 PMC4704385

[B5] Balamurugan K. (2016). HIF-1 at the crossroads of hypoxia, inflammation, and cancer. Int. J. Cancer 138, 1058–1066. 10.1002/ijc.29519 25784597 PMC4573780

[B6] Barcellos-Hoff M. H. Lyden D. Wang T. C. (2013). The evolution of the cancer niche during multistage carcinogenesis. Nat. Rev. Cancer 13, 511–518. 10.1038/nrc3536 23760023

[B7] Bassères D. S. Baldwin A. S. (2006). Nuclear factor-kappaB and inhibitor of kappaB kinase pathways in oncogenic initiation and progression. Oncogene 25, 6817–6830. 10.1038/sj.onc.1209942 17072330

[B8] BelAiba R. S. Bonello S. Zähringer C. Schmidt S. Hess J. Kietzmann T. (2007). Hypoxia up-regulates hypoxia-inducible factor-1alpha transcription by involving phosphatidylinositol 3-kinase and nuclear factor kappaB in pulmonary artery smooth muscle cells. Mol. Biol. Cell. 18, 4691–4697. 10.1091/mbc.E07-04-0391 17898080 PMC2096613

[B9] Ben-Neriah Y. Karin M. (2011). Inflammation meets cancer, with NF-κB as the matchmaker. Nat. Immunol. 12, 715–723. 10.1038/ni.2060 21772280

[B10] Benjamin D. Robay D. Hindupur S. K. Pohlmann J. Colombi M. El-Shemerly M. Y. (2018). Dual inhibition of the lactate transporters MCT1 and MCT4 is synthetic lethal with metformin due to NAD+ depletion in cancer cells. Cell. Rep. 25, 3047–3058. 10.1016/j.celrep.2018.11.043 30540938 PMC6302548

[B11] Bensaad K. Favaro E. Lewis C. A. Peck B. Lord S. Collins J. M. (2014). Fatty acid uptake and lipid storage induced by HIF-1α contribute to cell growth and survival after hypoxia-reoxygenation. Cell. Rep. 9, 349–365. 10.1016/j.celrep.2014.08.056 25263561

[B12] Bonavia R. Inda M. M. Vandenberg S. Cheng S. Y. Nagane M. Hadwiger P. (2012). EGFRvIII promotes glioma angiogenesis and growth through the NF-B, interleukin-8 pathway. Oncogene 31, 4054–4066. 10.1038/onc.2011.563 22139077 PMC3537826

[B13] Bonora M. Patergnani S. Rimessi A. de Marchi E. Suski J. M. Bononi A. (2012). ATP synthesis and storage. Purinergic Signal 8, 343–357. 10.1007/s11302-012-9305-8 22528680 PMC3360099

[B14] Bosch-Marce M. Okuyama H. Wesley J. B. Sarkar K. Kimura H. Liu Y. V. (2007). Effects of aging and hypoxia-inducible factor-1 activity on angiogenic cell mobilization and recovery of perfusion after limb ischemia. Circ. Res. 101, 1310–1318. 10.1161/CIRCRESAHA.107.153346 17932327

[B15] Brahimi-Horn M. C. Pouysségur J. (2007). Harnessing the hypoxia-inducible factor in cancer and ischemic disease. Biochem. Pharmacol. 73, 450–457. 10.1016/j.bcp.2006.10.013 17101119

[B16] Brisson L. Bański P. Sboarina M. Dethier C. Danhier P. Fontenille M. J. (2016). Lactate dehydrogenase B controls lysosome activity and autophagy in cancer. Cancer Cell. 30, 418–431. 10.1016/j.ccell.2016.08.005 27622334

[B17] Brooks G. A. (2018). The science and translation of lactate shuttle theory. Cell. Metab. 27, 757–785. 10.1016/j.cmet.2018.03.008 29617642

[B18] Bruick R. K. McKnight S. L. (2001). A conserved family of prolyl-4-hydroxylases that modify HIF. Science 294, 1337–1340. 10.1126/science.1066373 11598268

[B19] Büchler P. Reber H. A. Tomlinson J. S. Hankinson O. Kallifatidis G. Friess H. (2009). Transcriptional regulation of urokinase-type plasminogen activator receptor by hypoxia-inducible factor 1 is crucial for invasion of pancreatic and liver cancer. Neoplasia 11, 196–206. 10.1593/neo.08734 19177204 PMC2631144

[B20] Bui T. Thompson C. B. (2006). Cancer’s sweet tooth. Cancer Cell. 9, 419–420. 10.1016/j.ccr.2006.05.012 16766260

[B21] Campillo N. Falcones B. Otero J. Colina R. Gozal D. Navajas D. (2019). Differential oxygenation in tumor microenvironment modulates macrophage and cancer cell crosstalk: Novel experimental setting and proof of concept. Front. Oncol. 9, 43. 10.3389/fonc.2019.00043 30788287 PMC6373430

[B22] Ceradini D. J. Kulkarni A. R. Callaghan M. J. Tepper O. M. Bastidas N. Kleinman M. E. (2004). Progenitor cell trafficking is regulated by hypoxic gradients through HIF-1 induction of SDF-1. Nat. Med. 10, 858–864. 10.1038/nm1075 15235597

[B23] Chan D. A. Kawahara T. L. A. Sutphin P. D. Chang H. Y. Chi J. T. Giaccia A. J. (2009). Tumor vasculature is regulated by PHD2-mediated angiogenesis and bone marrow-derived cell recruitment. Cancer Cell. 15, 527–538. 10.1016/j.ccr.2009.04.010 19477431 PMC2846696

[B24] Chandel N. S. (2015). Also from cold spring harbor laboratory press cell survival and cell death mitochondria MYC and the pathway to cancer protein homeostasis quickstart molecular biology signaling by receptor tyrosine kinases signal transduction type 1 diabetes yeast intermediary metabolism. Available at: www.cshlpress.org (Accessed March 17, 2021).

[B25] Chandel N. S. (2021). Glycolysis. Cold Spring Harb. Perspect. Biol. 13, a040535. 10.1101/cshperspect.a040535 33941515 PMC8091952

[B26] Chen L. Deng H. Cui H. Fang J. Zuo Z. Deng J. (2018). Inflammatory responses and inflammation-associated diseases in organs. Oncotarget 9, 7204–7218. 10.18632/oncotarget.23208 29467962 PMC5805548

[B27] Christensen K. Aaberg-Jessen C. Andersen C. Goplen D. Bjerkvig R. Kristensen B. W. (2010). Immunohistochemical expression of stem cell, endothelial cell, and chemosensitivity markers in primary glioma spheroids cultured in serum-containing and serum-free medium. Neurosurgery 66, 933–947. 10.1227/01.NEU.0000368393.45935.46 20404698

[B28] Cockman M. E. Lancaster D. E. Stolze I. P. Hewitson K. S. McDonough M. A. Coleman M. L. (2006). Posttranslational hydroxylation of ankyrin repeats in IkappaB proteins by the hypoxia-inducible factor (HIF) asparaginyl hydroxylase, factor inhibiting HIF (FIH). Proc. Natl. Acad. Sci. U. S. A. 103, 14767–14772. 10.1073/pnas.0606877103 17003112 PMC1578504

[B29] Collins T. Read M. A. Neish A. S. Whitley M. Z. Thanos D. Maniatis T. (1995). Transcriptional regulation of endothelial cell adhesion molecules: NF-Kappa B and cytokine-inducible enhancers. FASEB J. Off. Publ. Fed. Am. Soc. Exp. Biol. 9, 899–909. 10.1096/fasebj.9.10.7542214 7542214

[B210] Cooper G. M. Hausman R. E. (2007). The development and causes of cancer. Cell A Mol. Approach, 743. Available at: http://www.ncbi.nlm.nih.gov/books/NBK9963/ [Accessed May 16, 2021].

[B30] Costa C. Incio J. Soares R. (2007). Angiogenesis and chronic inflammation: Cause or consequence? Angiogenesis 10, 149–166. 10.1007/s10456-007-9074-0 17457680

[B31] Cummins E. P. Berra E. Comerford K. M. Ginouves A. Fitzgerald K. T. Seeballuck F. (2006). Prolyl hydroxylase-1 negatively regulates IkappaB kinase-beta, giving insight into hypoxia-induced NFkappaB activity. Proc. Natl. Acad. Sci. U. S. A. 103, 18154–18159. 10.1073/pnas.0602235103 17114296 PMC1643842

[B32] Currie E. Schulze A. Zechner R. Walther T. C. Farese R. V. (2013). Cellular fatty acid metabolism and cancer. Cell. Metab. 18, 153–161. 10.1016/j.cmet.2013.05.017 23791484 PMC3742569

[B33] Däster S. Amatruda N. Calabrese D. Ivanek R. Turrini E. Droeser R. A. (2017). Induction of hypoxia and necrosis in multicellular tumor spheroids is associated with resistance to chemotherapy treatment. Oncotarget 8, 1725–1736. 10.18632/oncotarget.13857 27965457 PMC5352092

[B34] Dayan F. Monticelli M. Pouysségur J. Pécou E. (2009). Gene regulation in response to graded hypoxia: The non-redundant roles of the oxygen sensors PHD and FIH in the HIF pathway. J. Theor. Biol. 259, 304–316. 10.1016/j.jtbi.2009.03.009 19298827

[B35] Dayan F. Roux D. Brahimi-Horn M. C. Pouyssegur J. Mazure N. M. (2006). The oxygen sensor factor-inhibiting hypoxia-inducible factor-1 controls expression of distinct genes through the bifunctional transcriptional character of hypoxia-inducible factor-1alpha. Cancer Res. 66, 3688–3698. 10.1158/0008-5472.CAN-05-4564 16585195

[B36] De Saedeleer C. J. Copetti T. Porporato P. E. Verrax J. Feron O. Sonveaux P. (2012). Lactate activates HIF-1 in oxidative but not in warburg-phenotype human tumor cells. PLoS One 7, e46571. 10.1371/journal.pone.0046571 23082126 PMC3474765

[B37] DeBerardinis R. J. Mancuso A. Daikhin E. Nissim I. Yudkoff M. Wehrli S. (2007). Beyond aerobic glycolysis: Transformed cells can engage in glutamine metabolism that exceeds the requirement for protein and nucleotide synthesis. Proc. Natl. Acad. Sci. U. S. A. 104, 19345–19350. 10.1073/pnas.0709747104 18032601 PMC2148292

[B38] DeBerardinis R. J. Sayed N. Ditsworth D. Thompson C. B. (2008). Brick by brick: Metabolism and tumor cell growth. Curr. Opin. Genet. Dev. 18, 54–61. 10.1016/j.gde.2008.02.003 18387799 PMC2476215

[B39] Delhase M. Hayakawa M. Chen Y. Karin M. (1999). Positive and negative regulation of IkappaB kinase activity through IKKbeta subunit phosphorylation. Science 284, 309–313. 10.1126/science.284.5412.309 10195894

[B40] Déry M. A. C. Michaud M. D. Richard D. E. (2005). Hypoxia-inducible factor 1: Regulation by hypoxic and non-hypoxic activators. Int. J. Biochem. Cell. Biol. 37, 535–540. 10.1016/j.biocel.2004.08.012 15618010

[B41] Devi U. Singh M. Roy S. Gupta P. S. Ansari M. N. Saeedan A. S. (2019a). Activation of prolyl hydroxylase-2 for stabilization of mitochondrial stress along with simultaneous downregulation of HIF-1α/FASN in ER + breast cancer subtype. Cell. biochem. Funct. 37, 216–227. 10.1002/cbf.3389 30950543

[B42] Devi U. Singh M. Roy S. Tripathi A. C. Gupta P. S. Saraf S. K. (2019b). PHD-2 activation: A novel strategy to control HIF-1α and mitochondrial stress to modulate mammary gland pathophysiology in ER+ subtype. Naunyn. Schmiedeb. Arch. Pharmacol. 392, 1239–1256. 10.1007/s00210-019-01658-7 31154466

[B43] Dhup S. Kumar Dadhich R. Ettore Porporato P. Sonveaux P. (2012). Multiple biological activities of lactic acid in cancer: Influences on tumor Growth,Angiogenesis and metastasis. Curr. Pharm. Des. 18, 1319–1330. 10.2174/138161212799504902 22360558

[B44] Disis M. L. (2010). Immune regulation of cancer. J. Clin. Oncol. 28, 4531–4538. 10.1200/JCO.2009.27.2146 20516428 PMC3041789

[B45] Doherty J. R. Cleveland J. L. (2013). Targeting lactate metabolism for cancer therapeutics. J. Clin. Invest. 123, 3685–3692. 10.1172/JCI69741 23999443 PMC3754272

[B46] Dolcet X. Llobet D. Pallares J. Matias-Guiu X. (2005). NF-kB in development and progression of human cancer. Virchows Arch. 446, 475–482. 10.1007/s00428-005-1264-9 15856292

[B47] Dong Z. Wang J. Z. Yu F. Venkatachalam M. A. (2003). Apoptosis-resistance of hypoxic cells: Multiple factors involved and a role for IAP-2. Am. J. Pathol. 163, 663–671. 10.1016/S0002-9440(10)63693-0 12875985 PMC1868200

[B48] Dorrington M. G. Fraser I. D. C. (2019). NF-κB signaling in macrophages: Dynamics, crosstalk, and signal integration. Front. Immunol. 10, 705. 10.3389/fimmu.2019.00705 31024544 PMC6465568

[B49] Dupuy F. Tabariès S. Andrzejewski S. Dong Z. Blagih J. Annis M. G. (2015). PDK1-dependent metabolic reprogramming dictates metastatic potential in breast cancer. Cell. Metab. 22, 577–589. 10.1016/j.cmet.2015.08.007 26365179

[B50] Epstein T. Xu L. Gillies R. J. Gatenby R. A. (2014). Separation of metabolic supply and demand: Aerobic glycolysis as a normal physiological response to fluctuating energetic demands in the membrane. Cancer Metab. 2, 7. 10.1186/2049-3002-2-7 24982758 PMC4060846

[B51] Erez N. Truitt M. Olson P. Hanahan D. (2010). Cancer-associated fibroblasts are activated in incipient neoplasia to orchestrate tumor-promoting inflammation in an NF-kappaB-Dependent manner. Cancer Cell. 17, 135–147. 10.1016/j.ccr.2009.12.041 20138012

[B52] Fan J. Hitosugi T. Chung T.-W. Xie J. Ge Q. Gu T.-L. (2011). Tyrosine phosphorylation of lactate dehydrogenase A is important for NADH/NAD+ redox homeostasis in cancer cells. Mol. Cell. Biol. 31, 4938–4950. 10.1128/mcb.06120-11 21969607 PMC3233034

[B211] Fares J. Fares M. Y. Khachfe H. H. Salhab H. A. Fares Y. (2020). Molecular principles of metastasis: a hallmark of cancer revisited. Signal Transduct. Target. Ther. 5, 1–17. 10.1038/s41392-020-0134-x 32296047 PMC7067809

[B53] Favaro E. Bensaad K. Chong M. G. Tennant D. A. Ferguson D. J. P. Snell C. (2012). Glucose utilization via glycogen phosphorylase sustains proliferation and prevents premature senescence in cancer cells. Cell. Metab. 16, 751–764. 10.1016/j.cmet.2012.10.017 23177934

[B54] Fearon E. R. Vogelstein B. (1990). A genetic model for colorectal tumorigenesis. Cell. 61, 759–767. 10.1016/0092-8674(90)90186-I 2188735

[B55] Feron O. (2009). Pyruvate into lactate and back: From the Warburg effect to symbiotic energy fuel exchange in cancer cells. Radiother. Oncol. 92, 329–333. 10.1016/j.radonc.2009.06.025 19604589

[B56] Forsythe J. A. Jiang B. H. Iyer N. V. Agani F. Leung S. W. Koos R. D. (1996). Activation of vascular endothelial growth factor gene transcription by hypoxia-inducible factor 1. Mol. Cell. Biol. 16, 4604–4613. 10.1128/mcb.16.9.4604 8756616 PMC231459

[B57] Freedman S. J. Sun Z. Y. J. Poy F. Kung A. L. Livingston D. M. Wagner G. (2002). Structural basis for recruitment of CBP/p300 by hypoxia-inducible factor-1 alpha. Proc. Natl. Acad. Sci. U. S. A. 99, 5367–5372. 10.1073/pnas.082117899 11959990 PMC122775

[B58] Fulda S. (2014). Molecular pathways: Targeting inhibitor of apoptosis proteins in cancer-from molecular mechanism to therapeutic application. Clin. Cancer Res. 20, 289–295. 10.1158/1078-0432.CCR-13-0227 24270683

[B216] Fu X. Dai Z. Song K. Zhang Z. Zhou Z. Zhou S. (2015). Macrophage-secreted IL-8 induces epithelial-mesenchymal transition in hepatocellular carcinoma cells by activating the JAK2 / STAT3 / Snail pathway. 587–596. 10.3892/ijo.2014.2761 25405790

[B59] Furuta E. Pai S. K. Zhan R. Bandyopadhyay S. Watabe M. Mo Y. Y. (2008). Fatty acid synthase gene is up-regulated by hypoxia via activation of Akt and sterol regulatory element binding protein-1. Cancer Res. 68, 1003–1011. 10.1158/0008-5472.CAN-07-2489 18281474

[B60] Galluzzi L. Morselli E. Kepp O. Vitale I. Rigoni A. Vacchelli E. (2010). Mitochondrial gateways to cancer. Mol. Asp. Med. 31, 1–20. 10.1016/j.mam.2009.08.002 19698742

[B61] Gatenby R. A. Gillies R. J. (2004). Why do cancers have high aerobic glycolysis? Nat. Rev. Cancer 4, 891–899. 10.1038/nrc1478 15516961

[B62] Gatenby R. A. Smallbone K. Maini P. K. Rose F. Averill J. Nagle R. B. (2007). Cellular adaptations to hypoxia and acidosis during somatic evolution of breast cancer. Br. J. Cancer 97, 646–653. 10.1038/sj.bjc.6603922 17687336 PMC2360372

[B63] Gerald D. Berra E. Frapart Y. M. Chan D. A. Giaccia A. J. Mansuy D. (2004). JunD reduces tumor angiogenesis by protecting cells from oxidative stress. Cell. 118, 781–794. 10.1016/j.cell.2004.08.025 15369676

[B64] Gerber H. P. Dixit V. Ferrara N. (1998). Vascular endothelial growth factor induces expression of the antiapoptotic proteins Bcl-2 and A1 in vascular endothelial cells. J. Biol. Chem. 273, 13313–13316. 10.1074/jbc.273.21.13313 9582377

[B65] Ghosh G. Van Duyne G. Ghosh S. Sigler P. B. (1995). Structure of nf-κb p50 homodimer bound to a κb site. Nature 373, 303–310. 10.1038/373303a0 7530332

[B66] Gilmore T. D. (2006). Introduction to NF-kappaB: Players, pathways, perspectives. Oncogene 25, 6680–6684. 10.1038/sj.onc.1209954 17072321

[B67] Goetze K. Walenta S. Ksiazkiewicz M. Kunz-Schughart L. A. Mueller-Klieser W. (2011). Lactate enhances motility of tumor cells and inhibits monocyte migration and cytokine release. Int. J. Oncol. 39, 453–463. 10.3892/ijo.2011.1055 21617859

[B68] Greten F. R. Eckmann L. Greten T. F. Park J. M. Li Z. W. Egan L. J. (2004). IKKbeta links inflammation and tumorigenesis in a mouse model of colitis-associated cancer. Cell. 118, 285–296. 10.1016/j.cell.2004.07.013 15294155

[B69] Grimes D. R. Kelly C. Bloch K. Partridge M. (2014). A method for estimating the oxygen consumption rate in multicellular tumour spheroids. J. R. Soc. Interface 11, 20131124. 10.1098/rsif.2013.1124 24430128 PMC3899881

[B70] Grivennikov S. I. Greten F. R. Karin M. (2010). Immunity, inflammation, and cancer. Cell. 140, 883–899. 10.1016/j.cell.2010.01.025 20303878 PMC2866629

[B71] Gu Y. Z. Moran S. M. Hogenesch J. B. Wartman L. Bradfield C. A. (1998). Molecular characterization and chromosomal localization of a third α-class hypoxia inducible factor subunit, HIF3α. Gene Expr. 7, 205–213. Available at: http://dot (Accessed May 17, 2021). 9840812 PMC6151950

[B72] Gudkov A. V. Gurova K. V. Komarova E. A. (2011). Inflammation and p53: A tale of two stresses. Genes. Cancer 2, 503–516. 10.1177/1947601911409747 21779518 PMC3135644

[B73] Guppy M. Greiner E. Brand K. (1993). The role of the Crabtree effect and an endogenous fuel in the energy metabolism of resting and proliferating thymocytes. Eur. J. Biochem. 212, 95–99. 10.1111/j.1432-1033.1993.tb17637.x 8444168

[B74] Gupta S. C. Singh R. Pochampally R. Watabe K. Mo Y. Y. (2014). Acidosis promotes invasiveness of breast cancer cells through ROS-AKT-NF-κB pathway. Oncotarget 5, 12070–12082. 10.18632/oncotarget.2514 25504433 PMC4322981

[B75] Halestrap A. P. (2013). The SLC16 gene family-Structure, role and regulation in health and disease. Mol. Asp. Med. 34, 337–349. 10.1016/j.mam.2012.05.003 23506875

[B76] Helbig G. Christopherson K. W. Bhat-Nakshatri P. Kumar S. Kishimoto H. Miller K. D. (2003). NF-kappaB promotes breast cancer cell migration and metastasis by inducing the expression of the chemokine receptor CXCR4. J. Biol. Chem. 278, 21631–21638. 10.1074/jbc.M300609200 12690099

[B77] Höckel M. Vaupel P. (2001). Tumor hypoxia: Definitions and current clinical, biologic, and molecular aspects. J. Natl. Cancer Inst. 93, 266–276. 10.1093/jnci/93.4.266 11181773

[B217] Hospital B. F. (2018). TNF- α promotes colon cancer cell migration and invasion by upregulating TROP-2. 3820 –3827. 10.3892/ol.2018.7735 PMC5796310 29467899

[B78] Hu C. J. Sataur A. Wang L. Chen H. Simon M. C. (2007). The N-terminal transactivation domain confers target gene specificity of hypoxia-inducible factors HIF-1alpha and HIF-2alpha. Mol. Biol. Cell. 18, 4528–4542. 10.1091/mbc.E06-05-0419 17804822 PMC2043574

[B79] Huang S. Robinson J. B. DeGuzman A. Bucana C. D. Fidler I. J. (2000). Blockade of nuclear factor-kappaB signaling inhibits angiogenesis and tumorigenicity of human ovarian cancer cells by suppressing expression of vascular endothelial growth factor and interleukin 8. Cancer Res. 60, 5334–5339. 11034066

[B215] Hung K. E. Datz C. Feng Y. Fearon E. R. (2012). products drive IL-23 / IL-17-mediated tumour growth. 10.1038/nature11465 PMC3601659 23034650

[B80] Huber M. A. Azoitei N. Baumann B. Grünert S. Sommer A. Pehamberger H. (2004). NF-kappaB is essential for epithelial-mesenchymal transition and metastasis in a model of breast cancer progression. J. Clin. Invest. 114, 569–581. 10.1172/jci21358 15314694 PMC503772

[B81] Jiang B. H. Rue E. Wang G. L. Roe R. Semenza G. L. (1996). Dimerization, DNA binding, and transactivation properties of hypoxia-inducible factor 1. J. Biol. Chem. 271, 17771–17778. 10.1074/jbc.271.30.17771 8663540

[B82] Johnson L. N. Noble M. E. M. Owen D. J. (1996). Active and inactive protein kinases: Structural basis for regulation. Cell. 85, 149–158. 10.1016/S0092-8674(00)81092-2 8612268

[B83] Kaelin W. G. Ratcliffe P. J. (2008). Oxygen sensing by metazoans: The central role of the HIF hydroxylase pathway. Mol. Cell. 30, 393–402. 10.1016/j.molcel.2008.04.009 18498744

[B84] Kaelin W. G. (2003). The von Hippel-lindau gene, kidney cancer, and oxygen sensing. J. Am. Soc. Nephrol. 14, 2703–2711. 10.1097/01.ASN.0000092803.69761.41 14569079

[B85] Kalluri R. (2016). The biology and function of fibroblasts in cancer. Nat. Rev. Cancer 16, 582–598. 10.1038/nrc.2016.73 27550820

[B86] Kaltschmidt B. Greiner J. F. W. Kadhim H. M. Kaltschmidt C. (2018). Subunit-specific role of NF-κB in cancer. Biomedicines 6, 44–12. 10.3390/biomedicines6020044 29673141 PMC6027219

[B87] Kamphorst J. J. Chung M. K. Fan J. Rabinowitz J. D. (2014). Quantitative analysis of acetyl-CoA production in hypoxic cancer cells reveals substantial contribution from acetate. Cancer Metab. 2, 23. 10.1186/2049-3002-2-23 25671109 PMC4322440

[B88] Karagiota A. Kourti M. Simos G. Mylonis I. (2019). HIF-1α-derived cell-penetrating peptides inhibit ERK-dependent activation of HIF-1 and trigger apoptosis of cancer cells under hypoxia. Cell. Mol. Life Sci. 76, 809–825. 10.1007/s00018-018-2985-7 30535970 PMC11105304

[B213] Karin M. Lawrence T. Nizet V. (2006). Innate immunity gone awry: Linking microbial infections to chronic inflammation and cancer. Cell 124, 823–835. 10.1016/j.cell.2006.02.016 16497591

[B89] Keith B. Johnson R. S. Simon M. C. (2012). HIF1 α and HIF2 α: sibling rivalry in hypoxic tumour growth and progression. Nat. Rev. Cancer 12, 9–22. 10.1038/nrc3183 PMC3401912 22169972

[B90] Kelly B. D. Hackett S. F. Hirota K. Oshima Y. Cai Z. Berg-Dixon S. (2003). Cell type-specific regulation of angiogenic growth factor gene expression and induction of angiogenesis in nonischemic tissue by a constitutively active form of hypoxia-inducible factor 1. Circ. Res. 93, 1074–1081. 10.1161/01.RES.0000102937.50486.1B 14576200

[B91] Kennedy F. G. Jones D. P. (1986). Oxygen dependence of mitochondrial function in isolated rat cardiac myocytes. Am. J. Physiol. - Cell. Physiol. 250, C374–C383. 10.1152/ajpcell.1986.250.3.c374 3953808

[B220] Kidane D. Chae W. J. Czochor J. Eckert K. A. Glazer P. M. Bothwell A. L. M. (2014). Interplay between DNA repair and inflammation, and the link to cancer. Crit. Rev. Biochem. Mol. Biol. 49, 116–139. 10.3109/10409238.2013.875514 24410153 PMC4300235

[B92] Kim J. W. Tchernyshyov I. Semenza G. L. Dang C. V. (2006). HIF-1-mediated expression of pyruvate dehydrogenase kinase: A metabolic switch required for cellular adaptation to hypoxia. Cell. Metab. 3, 177–185. 10.1016/j.cmet.2006.02.002 16517405

[B93] Kiraly O. Gong G. Olipitz W. Muthupalani S. Engelward B. P. (2015). Inflammation-induced cell proliferation potentiates DNA damage-induced mutations *in vivo* . PLoS Genet. 11, e1004901–e1004924. 10.1371/journal.pgen.1004901 25647331 PMC4372043

[B94] Knies N. Alankus B. Weilemann A. Tzankov A. Brunner K. Ruff T. (2015). Lymphomagenic CARD11/BCL10/MALT1 signaling drives malignant B-cell proliferation via cooperative NF-κB and JNK activation. Proc. Natl. Acad. Sci. U. S. A. 112, E7230–E7238. 10.1073/pnas.1507459112 26668357 PMC4702996

[B95] Koh M. Y. Powis G. (2012). Passing the baton: The HIF switch. Trends biochem. Sci. 37, 364–372. 10.1016/j.tibs.2012.06.004 22818162 PMC3433036

[B96] Koivunen P. Hirsilä M. Günzler V. Kivirikko K. I. Myllyharju J. (2004). Catalytic properties of the asparaginyl hydroxylase (FIH) in the oxygen sensing pathway are distinct from those of its prolyl 4-hydroxylases. J. Biol. Chem. 279, 9899–9904. 10.1074/jbc.M312254200 14701857

[B97] Koong A. C. Chen E. Y. Giaccia A. J. (1994). Hypoxia causes the activation of nuclear factor κB through the phosphorylation of IκBα on tyrosine residues. Cancer Res. 54, 1425–1430. 8137243

[B98] Kourti M. Ikonomou G. Giakoumakis N. N. Rapsomaniki M. A. Landegren U. Siniossoglou S. (2015). CK1δ restrains lipin-1 induction, lipid droplet formation and cell proliferation under hypoxia by reducing HIF-1α/ARNT complex formation. Cell. Signal. 27, 1129–1140. 10.1016/j.cellsig.2015.02.017 25744540 PMC4390155

[B99] Krishnamachary B. Zagzag D. Nagasawa H. Rainey K. Okuyama H. Baek J. H. (2006). Hypoxia-inducible factor-1-dependent repression of E-cadherin in von Hippel-Lindau tumor suppressor-null renal cell carcinoma mediated by TCF3, ZFHX1A, and ZFHX1B. Cancer Res. 66, 2725–2731. 10.1158/0008-5472.CAN-05-3719 16510593

[B100] Krishnan J. Suter M. Windak R. Krebs T. Felley A. Montessuit C. (2009). Activation of a HIF1alpha-PPARgamma axis underlies the integration of glycolytic and lipid anabolic pathways in pathologic cardiac hypertrophy. Cell. Metab. 9, 512–524. 10.1016/j.cmet.2009.05.005 19490906

[B219] Kryston T. B. Georgiev A. B. Pissis P. Georgakilas A. G. (2011). Role of oxidative stress and DNA damage in human carcinogenesis. Mutat. Res. - Fundam. Mol. Mech. Mutagen. 711, 193–201. 10.1016/j.mrfmmm.2010.12.016 21216256

[B101] Landén N. X. Li D. Ståhle M. (2016). Transition from inflammation to proliferation: A critical step during wound healing. Cell. Mol. Life Sci. 73, 3861–3885. 10.1007/s00018-016-2268-0 27180275 PMC5021733

[B102] Lando D. Peet D. J. Gorman J. J. Whelan D. A. Whitelaw M. L. Bruick R. K. (2002). FIH-1 is an asparaginyl hydroxylase enzyme that regulates the transcriptional activity of hypoxia-inducible factor. Genes. Dev. 16, 1466–1471. 10.1101/gad.991402 12080085 PMC186346

[B103] Ledoux A. C. Perkins N. D. (2014). NF-κB and the cell cycle. Biochem. Soc. Trans. 42, 76–81. 10.1042/BST20130156 24450631

[B104] Lee G. Won H. S. Lee Y. M. Choi J. W. Oh T. I. Jang J. H. (2016). Oxidative dimerization of PHD2 is responsible for its inactivation and contributes to metabolic reprogramming via HIF-1α activation. Sci. Rep. 6, 18928. 10.1038/srep18928 26740011 PMC4703963

[B105] Lee K. A. Zhang H. Qian D. Z. Rey S. Liu J. O. Semenza G. L. (2009). Acriflavine inhibits HIF-1 dimerization, tumor growth, and vascularization. Proc. Natl. Acad. Sci. U. S. A. 106, 17910–17915. 10.1073/pnas.0909353106 19805192 PMC2764905

[B212] León X. Bothe C. García J. Parreño M. Alcolea S. Quer M. Expression of IL-1α correlates with distant metastasis in patients with head and neck squamous cell carcinoma. Oncotarget 6 (35), 37398. 26460957 10.18632/oncotarget.6054 PMC4741937

[B106] Ling L. Cao Z. Goeddel D. V. (1998). NF-kappaB-inducing kinase activates IKK-alpha by phosphorylation of Ser-176. Proc. Natl. Acad. Sci. U. S. A. 95, 3792–3797. 10.1073/pnas.95.7.3792 9520446 PMC19916

[B107] Liou G. Storz P. (2010). Reactive oxygen species in cancer. Free Radic. Res. 44, 479–496. 10.3109/10715761003667554 20370557 PMC3880197

[B108] Liston P. Fong W. G. Korneluk R. G. (2003). The inhibitors of apoptosis: There is more to life than Bcl2. Oncogene 22, 8568–8580. 10.1038/sj.onc.1207101 14634619

[B109] Liu F. Xia Y. Parker A. S. Verma I. M. (2012). IKK biology. Immunol. Rev. 246, 239–253. 10.1111/j.1600-065X.2012.01107.x 22435559 PMC3311052

[B110] Maisonpierre P. C. Suri C. Jones P. F. Bartunkova S. Wiegand S. J. Radziejewski C. (1997). Angiopoietin-2, a natural antagonist for Tie2 that disrupts *in vivo* angiogenesis. Science 277, 55–60. 10.1126/science.277.5322.55 9204896

[B111] Malek S. Huang D. B. Huxford T. Ghosh S. Ghosh G. (2003). X-ray crystal structure of an IkappaBbeta x NF-kappaB p65 homodimer complex. J. Biol. Chem. 278, 23094–23100. 10.1074/jbc.M301022200 12686541

[B112] Marienfeld R. May M. J. Berberich I. Serfling E. Ghosh S. Neumann M. (2003). RelB forms transcriptionally inactive complexes with RelA/p65. J. Biol. Chem. 278, 19852–19860. 10.1074/jbc.M301945200 12657634

[B113] Martínez-Reyes I. Chandel N. S. (2020). Mitochondrial TCA cycle metabolites control physiology and disease. Nat. Commun. 11, 102. 10.1038/s41467-019-13668-3 31900386 PMC6941980

[B114] Martins G. R. Gelaleti G. B. Moschetta M. G. Maschio-signorini L. B. Ap D. Campos P. D. (2016). Proinflammatory and anti-inflammatory cytokines mediated by NF-κB factor as prognostic markers in mammary tumors. Mediat. Inflamm. 2016, 9512743. 10.1155/2016/9512743 PMC4771900 26989335

[B115] Mashimo T. Pichumani K. Vemireddy V. Hatanpaa K. J. Singh D. K. Sirasanagandla S. (2014). Acetate is a bioenergetic substrate for human glioblastoma and brain metastases. Cell. 159, 1603–1614. 10.1016/j.cell.2014.11.025 25525878 PMC4374602

[B116] Masoud G. N. Li W. (2015). HIF-1α pathway: Role, regulation and intervention for cancer therapy. Acta Pharm. Sin. B 5, 378–389. 10.1016/j.apsb.2015.05.007 26579469 PMC4629436

[B117] Maxwell P. H. Wlesener M. S. Chang G. W. Clifford S. C. Vaux E. C. Cockman M. E. (1999). The tumour suppressor protein VHL targets hypoxia-inducible factors for oxygen-dependent proteolysis. Nature 399, 271–275. 10.1038/20459 10353251

[B118] McKeown S. R. (2014). Defining normoxia, physoxia and hypoxia in tumours - implications for treatment response. Br. J. Radiol. 87, 20130676. 10.1259/bjr.20130676 24588669 PMC4064601

[B119] McNeill L. A. Hewitson K. S. Claridge T. D. Seibel J. F. Horsfall L. E. Schofield C. J. (2002). Hypoxia-inducible factor asparaginyl hydroxylase (FIH-1) catalyses hydroxylation at the β-carbon of asparagine-803. Biochem. J. 367, 571–575. 10.1042/BJ20021162 12215170 PMC1222951

[B120] Mercurio F. Zhu H. Murray B. W. Shevchenko A. Bennett B. L. Li J. W. (1997). IKK-1 and IKK-2: Cytokine-activated IkappaB kinases essential for NF-kappaB activation. Science 278, 860–866. 10.1126/science.278.5339.860 9346484

[B121] Milane L. Duan Z. Amiji M. (2011). Role of hypoxia and glycolysis in the development of multi-drug resistance in human tumor cells and the establishment of an orthotopic multi-drug resistant tumor model in nude mice using hypoxic pre-conditioning. Cancer Cell. Int. 11, 3–16. 10.1186/1475-2867-11-3 21320311 PMC3045873

[B122] Mishra D. Banerjee D. (2019). Lactate dehydrogenases as metabolic links between tumor and stroma in the tumor microenvironment. Cancers (Basel) 11, 750. 10.3390/cancers11060750 31146503 PMC6627402

[B123] Moreadiths R. W. Lehningert A. L. (1984). The pathways of glutamate and glutamine oxidation by tumor cell mitochondria. Role of mitochondrial NAD(P)+-dependent malic enzyme. J. Biol. Chem. 259 (10), 6215–6221. 10.1016/S0021-9258(20)82128-0 6144677

[B124] Mukerjee S. Saeedan A. S. Ansari M. N. Singh M. (2021). Polyunsaturated fatty acids mediated regulation of membrane biochemistry and tumor cell membrane integrity. Membr. (Basel) 11, 479. 10.3390/membranes11070479 PMC8304949 34203433

[B125] Mylonis I. Sembongi H. Befani C. Liakos P. Siniossoglou S. Simos G. (2012). Hypoxia causes triglyceride accumulation by HIF-1-mediated stimulation of lipin 1 expression. J. Cell. Sci. 125, 3485–3493. 10.1242/jcs.106682 22467849 PMC3516382

[B126] Nakajima E. C. Van Houten B. (2013). Metabolic symbiosis in cancer: Refocusing the Warburg lens. Mol. Carcinog. 52, 329–337. 10.1002/mc.21863 22228080 PMC9972501

[B127] Nakayama K. Kataoka N. (2019). Regulation of gene expression under hypoxic conditions. Int. J. Mol. Sci. 20, 3278. 10.3390/ijms20133278 31277312 PMC6651685

[B128] Nakshatri H. Appaiah H. N. Anjanappa M. Gilley D. Tanaka H. Badve S. (2015). NF-κB-dependent and -independent epigenetic modulation using the novel anti-cancer agent DMAPT. Cell. Death Dis. 6, e1608. 10.1038/cddis.2014.569 25611383 PMC4669767

[B129] Nam S. Y. Ko Y. S. Jung J. Yoon J. Kim Y. H. Choi Y. J. (2011). A hypoxia-dependent upregulation of hypoxia-inducible factor-1 by nuclear factor-B promotes gastric tumour growth and angiogenesis. Br. J. Cancer 104, 166–174. 10.1038/sj.bjc.6606020 21119667 PMC3039796

[B130] Nguyen D. X. Bos P. D. Massagué J. (2009). Metastasis: From dissemination to organ-specific colonization. Nat. Rev. Cancer 9, 274–284. 10.1038/nrc2622 19308067

[B131] Oeckinghaus A. Hayden M. S. Ghosh S. (2011). Crosstalk in NF-κB signaling pathways. Nat. Immunol. 12, 695–708. 10.1038/ni.2065 21772278

[B132] Oliver K. M. Garvey J. F. Ng C. T. Veale D. J. Fearon U. Cummins E. P. (2009). Hypoxia activates NF-kappaB-dependent gene expression through the canonical signaling pathway. Antioxidants Redox Signal 11, 2057–2064. 10.1089/ars.2008.2400 19422287

[B133] Pan Y. Mansfield K. D. Bertozzi C. C. Rudenko V. Chan D. A. Giaccia A. J. (2007). Multiple factors affecting cellular redox status and energy metabolism modulate hypoxia-inducible factor prolyl hydroxylase activity *in vivo* and *in vitro* . Mol. Cell. Biol. 27, 912–925. 10.1128/mcb.01223-06 17101781 PMC1800695

[B134] Papandreou I. Krishna C. Kaper F. Cai D. Giaccia A. J. Denko N. C. (2005). Anoxia is necessary for tumor cell toxicity caused by a low-oxygen environment. Cancer Res. 65, 3171–3178. 10.1158/0008-5472.CAN-04-3395 15833847

[B135] Park M. Hong J. (2016). Roles of NF-κB in cancer and inflammatory diseases and their therapeutic approaches. Cells 5, 15. 10.3390/cells5020015 27043634 PMC4931664

[B136] Pavlova N. N. Thompson C. B. (2016). The emerging hallmarks of cancer metabolism. Cell. Metab. 23, 27–47. 10.1016/j.cmet.2015.12.006 26771115 PMC4715268

[B137] Perkins N. D. (2012). The diverse and complex roles of NF-κB subunits in cancer. Nat. Rev. Cancer 12, 121–132. 10.1038/nrc3204 22257950

[B138] Perman J. C. Boström P. Lindbom M. Lidberg U. StÅhlman M. Hägg D. (2011). The VLDL receptor promotes lipotoxicity and increases mortality in mice following an acute myocardial infarction. J. Clin. Invest. 121, 2625–2640. 10.1172/JCI43068 21670500 PMC3223818

[B139] Pescador N. Villar D. Cifuentes D. Garcia-Rocha M. Ortiz-Barahona A. Vazquez S. (2010). Hypoxia promotes glycogen accumulation through hypoxia inducible factor (HIF)-mediated induction of glycogen synthase 1. PLoS One 5, e9644. 10.1371/journal.pone.0009644 20300197 PMC2837373

[B140] Pfeiffer T. Schuster S. Bonhoeffer S. (2001). Undefined Cooperation and competition in the evolution of ATP-producing pathways. science 292, 504–507. Available at: https://science.sciencemag.org/content/292/5516/504.abstract (Accessed May 17, 2021). 10.1126/science.1058079 11283355

[B141] Pikarsky E. Porat R. M. Stein I. Abramovitch R. Amit S. Kasem S. (2004). NF- κB functions as a tumour promoter in inflammation-associated cancer. Nature 431, 461–466. 10.1038/nature02924 15329734

[B142] Pinheiro C. Longatto-Filho A. Azevedo-Silva J. Casal M. Schmitt F. C. Baltazar F. (2012). Role of monocarboxylate transporters in human cancers: State of the art. J. Bioenerg. Biomembr. 44, 127–139. 10.1007/s10863-012-9428-1 22407107

[B143] Pires B. R. B. Mencalha A. L. Ferreira G. M. de Souza W. F. Morgado-Díaz J. A. Maia A. M. (2017). NF-kappaB is involved in the regulation of EMT genes in breast cancer cells. PLoS One 12, e0169622. 10.1371/journal.pone.0169622 28107418 PMC5249109

[B144] Piret J. P. Mottet D. Raes M. Michiels C. (2002). Is HIF-1α a pro- or an anti-apoptotic protein? Biochem. Pharmacol. 64, 889–892. 10.1016/S0006-2952(02)01155-3 12213583

[B145] Pitot H. C. Goldsworthy T. Moran S. (1981). The natural history of carcinogenesis: Implications of experimental carcinogenesis in the Genesis of human cancer. J. Supramol. Struct. Cell. Biochem. 17, 133–146. 10.1002/jsscb.380170204 7033553

[B146] Psaila B. Lyden D. (2009). The metastatic niche: Adapting the foreign soil. Nat. Rev. Cancer 9, 285–293. 10.1038/nrc2621 19308068 PMC3682494

[B147] Qutub A. A. Popel A. S. (2006). A computational model of intracellular oxygen sensing by hypoxia-inducible factor HIF1 alpha. J. Cell. Sci. 119, 3467–3480. 10.1242/jcs.03087 16899821 PMC2129128

[B148] Rani S. Roy S. Singh M. Kaithwas G. (2022). Regulation of transactivation at C-tad domain of HIF-1α by factor-inhibiting HIF-1α (FIH-1): A potential target for therapeutic intervention in cancer. Oxid. Med. Cell. Longev. 2022, 2407223. 10.1155/2022/2407223 35592530 PMC9113874

[B149] Rao X. Duan X. Mao W. Li X. Li Z. Li Q. (2015). O-GlcNAcylation of G6PD promotes the pentose phosphate pathway and tumor growth. Nat. Commun. 6, 8468. 10.1038/ncomms9468 26399441 PMC4598839

[B150] Reinmuth N. Liu W. Jung Y. D. Ahmad S. A. Shaheen R. M. Fan F. (2001). Induction of VEGF in perivascular cells defines a potential paracrine mechanism for endothelial cell survival. FASEB J. 15, 1239–1241. 10.1096/fj.00-0693fje 11344100

[B151] Ren J. Wang Y. Gao Y. Mehta S. B. K. Lee C. G. L. (2011). FAT10 mediates the effect of TNF-α in inducing chromosomal instability. J. Cell. Sci. 124, 3665–3675. 10.1242/jcs.087403 22025632

[B152] Rey S. Semenza G. L. (2010). Hypoxia-inducible factor-1-dependent mechanisms of vascularization and vascular remodelling. Cardiovasc. Res. 86, 236–242. 10.1093/cvr/cvq045 20164116 PMC2856192

[B153] Riffle S. Hegde R. S. (2017). Modeling tumor cell adaptations to hypoxia in multicellular tumor spheroids. J. Exp. Clin. Cancer Res. 36, 102–110. 10.1186/s13046-017-0570-9 28774341 PMC5543535

[B154] Rocha S. (2007). Gene regulation under low oxygen: Holding your breath for transcription. Trends biochem. Sci. 32, 389–397. 10.1016/j.tibs.2007.06.005 17624786

[B155] Roy S. Rawat A. K. Sammi S. R. Devi U. Singh M. Gautam S. (2017). Alpha-linolenic acid stabilizes HIF-1 α and downregulates FASN to promote mitochondrial apoptosis for mammary gland chemoprevention. Oncotarget 8, 70049–70071. 10.18632/oncotarget.19551 29050261 PMC5642536

[B156] Roy S. Singh M. Rawat A. Devi U. Gautam S. Yadav R. K. (2018). GLA supplementation regulates PHD2 mediated hypoxia and mitochondrial apoptosis in DMBA induced mammary gland carcinoma. Int. J. Biochem. Cell. Biol. 96, 51–62. 10.1016/j.biocel.2018.01.011 29355756

[B157] Roy S. Singh M. Rawat A. Kumar D. Kaithwas G. (2020). Mitochondrial apoptosis and curtailment of hypoxia-inducible factor-1α/fatty acid synthase: A dual edge perspective of gamma linolenic acid in ER+ mammary gland cancer. Cell. biochem. Funct. 38, 591–603. 10.1002/cbf.3513 32207176

[B158] Roy S. Singh M. Sammi S. R. Pandey R. Kaithwas G. (2019). ALA-mediated biphasic downregulation of α-7nAchR/HIF-1α along with mitochondrial stress modulation strategy in mammary gland chemoprevention. J. Cell. Physiol. 234, 4015–4029. 10.1002/jcp.27168 30221357

[B159] Samanta D. Semenza G. L. (2018). Metabolic adaptation of cancer and immune cells mediated by hypoxia-inducible factors. Biochim. Biophys. Acta - Rev. Cancer 1870, 15–22. 10.1016/j.bbcan.2018.07.002 30006019

[B160] Schofield C. J. Ratcliffe P. J. (2004). Oxygen sensing by HIF hydroxylases. Nat. Rev. Mol. Cell. Biol. 5, 343–354. 10.1038/nrm1366 15122348

[B161] Schofield C. J. Ratcliffe P. J. (2005). Signalling hypoxia by HIF hydroxylases. Biochem. Biophys. Res. Commun. 338, 617–626. 10.1016/j.bbrc.2005.08.111 16139242

[B214] Schottenfeld D. Beebe-Dimmer J. (2006). Chronic Inflammation: A Common and Important Factor in the Pathogenesis of Neoplasia. CA Cancer J. Clin. 56, 69–83. 10.3322/canjclin.56.2.69 16514135

[B162] Schug Z. T. Peck B. Jones D. T. Zhang Q. Grosskurth S. Alam I. S. (2015). Acetyl-CoA synthetase 2 promotes acetate utilization and maintains cancer cell growth under metabolic stress. Cancer Cell. 27, 57–71. 10.1016/j.ccell.2014.12.002 25584894 PMC4297291

[B163] Semenza G. L. (2012). Hypoxia-inducible factors in physiology and medicine. Cell. 148, 399–408. 10.1016/j.cell.2012.01.021 22304911 PMC3437543

[B164] Semenza G. L. (2014). Oxygen sensing, hypoxia-inducible factors, and disease pathophysiology. Annu. Rev. Pathol. Mech. Dis. 9, 47–71. 10.1146/annurev-pathol-012513-104720 23937437

[B165] Semenza G. L. Jiang B. H. Leung S. W. Passantino R. Concordat J. P. Maire P. (1996). Hypoxia response elements in the aldolase A, enolase 1, and lactate dehydrogenase a gene promoters contain essential binding sites for hypoxia-inducible factor 1. J. Biol. Chem. 271, 32529–32537. 10.1074/jbc.271.51.32529 8955077

[B166] Sen R. Baltimore D. (1986). Multiple nuclear factors interact with the immunoglobulin enhancer sequences. Cell. 46, 705–716. 10.1016/0092-8674(86)90346-6 3091258

[B218] Seol M. A. Kim J. Oh K. Kim G. Seo M. W. Shin Y. (2019). Interleukin-7 Contributes to the Invasiveness of Prostate Cancer Cells by Promoting Epithelial – Mesenchymal Transition. Sci. Rep., 1, 6917–12. 10.1038/s41598-019-43294-4 PMC6502845 31061414

[B167] Shestov A. A. Liu X. Ser Z. Cluntun A. A. Hung Y. P. Huang L. (2014). Quantitative determinants of aerobic glycolysis identify flux through the enzyme GAPDH as a limiting step. Elife 3, 03342. 10.7554/eLife.03342 PMC4118620 25009227

[B168] Simon M.-P. Tournaire R. Pouyssegur J. (2008). The angiopoietin-2 gene of endothelial cells is up-regulated in hypoxia by a HIF binding site located in its first intron and by the central factors GATA-2 and Ets-1. J. Cell. Physiol. 217, 809–818. 10.1002/jcp.21558 18720385

[B169] Singh L. Aldosary S. Saeedan A. S. Ansari M. N. Kaithwas G. (2018a). Prolyl hydroxylase 2: A promising target to inhibit hypoxia-induced cellular metabolism in cancer cells. Drug Discov. Today 23, 1873–1882. 10.1016/j.drudis.2018.05.016 29772209

[B170] Singh L. Nair L. Kumar D. (2023). Hypoxia induced lactate acidosis modulates tumor microenvironment and lipid reprogramming to sustain the cancer cell survival. 1–18. doi: 10.3389/fonc.2023.1034205 PMC9906992 36761981

[B171] Singh L. Roy S. Kumar A. Rastogi S. Kumar D. Ansari M. N. (2021a). Repurposing combination therapy of voacamine with vincristine for downregulation of hypoxia-inducible factor-1α/fatty acid synthase Co-axis and prolyl hydroxylase-2 activation in ER+ mammary neoplasia. Front. Cell. Dev. Biol. 9, 736910–736919. 10.3389/fcell.2021.736910 34869321 PMC8637442

[B172] Singh L. Singh M. Rastogi S. Choudhary A. Kumar D. Raj R. (2021b). Effect of Voacamine upon inhibition of hypoxia induced fatty acid synthesis in a rat model of methyln-nitrosourea induced mammary gland carcinoma. BMC Mol. Cell. Biol. 22, 33–17. 10.1186/s12860-021-00371-9 34090331 PMC8180083

[B173] Singh M. Devi U. Roy S. Gupta P. S. Kaithwas G. (2018b). Chemical activation of prolyl hydroxylase-2 by BBAP-1 down regulates hypoxia inducible factor-1α and fatty acid synthase for mammary gland chemoprevention. RSC Adv. 8, 12848–12860. 10.1039/c8ra01239c 35541235 PMC9079607

[B174] Singh M. Devi U. Roy S. Gupta P. S. Saraf S. A. Kaithwas G. (2016). Prolyl hydroxylase mediated inhibition of fatty acid synthase to combat tumor growth in mammary gland carcinoma. Breast Cancer 23, 820–829. 10.1007/s12282-016-0683-6 26951539

[B175] Smith S. M. Lyu Y. L. Cai L. (2014). NF-κB affects proliferation and invasiveness of breast cancer cells by regulating CD44 expression. PLoS One 9, 106966. 10.1371/journal.pone.0106966 PMC4153718 25184276

[B176] Sonveaux P. Végran F. Schroeder T. Wergin M. C. Verrax J. Rabbani Z. N. (2008). Targeting lactate-fueled respiration selectively kills hypoxic tumor cells in mice. J. Clin. Invest. 118, 3930–3942. 10.1172/JCI36843 19033663 PMC2582933

[B177] Sowter H. M. Ratcliffe P. J. Watson P. Greenberg A. H. Harris A. L. (2001). HIF-1-dependent regulation of hypoxic induction of the cell death factors BNIP3 and NIX in human tumors 1. Available at: http://www.ncbi.nlm.nih.gov/SAGE (Accessed May 17, 2021). 11559532

[B178] Staller P. Sulitkova J. Lisztwan J. Moch H. Oakeley E. J. Krek W. (2003). Chemokine receptor CXCR4 downregulated by von Hippel-Lindau tumour suppressor pVHL. Nature 425, 307–311. 10.1038/nature01874 13679920

[B179] Stolze I. P. Tian Y. M. Appelhoff R. J. Turley H. Wykoff C. C. Gleadle J. M. (2004). Genetic analysis of the role of the asparaginyl hydroxylase factor inhibiting hypoxia-inducible factor (FIH) in regulating hypoxia-inducible factor (HIF) transcriptional target genes [corrected]. J. Biol. Chem. 279, 42719–42725. 10.1074/jbc.M406713200 15302861

[B180] Sun S. C. Chang J. H. Jin J. (2013). Regulation of nuclear factor-κB in autoimmunity. Trends Immunol. 34, 282–289. 10.1016/j.it.2013.01.004 23434408 PMC3664242

[B181] Sun S. C. (2011). Non-canonical NF-κB signaling pathway. Cell. Res. 21, 71–85. 10.1038/cr.2010.177 21173796 PMC3193406

[B182] Sun S. C. (2017). The non-canonical NF-κB pathway in immunity and inflammation. Nat. Rev. Immunol. 17, 545–558. 10.1038/nri.2017.52 28580957 PMC5753586

[B183] Suzuki H. Tomida A. Tsuruo T. (2001). Dephosphorylated hypoxia-inducible factor 1alpha as a mediator of p53-dependent apoptosis during hypoxia. Oncogene 20, 5779–5788. 10.1038/sj.onc.1204742 11593383

[B184] Takeda Y. Costa S. Delamarre E. Roncal C. Leite de Oliveira R. Squadrito M. L. (2011). Macrophage skewing by Phd2 haplodeficiency prevents ischaemia by inducing arteriogenesis. Nature 479, 122–126. 10.1038/nature10507 21983962 PMC4659699

[B185] Taylor C. T. Cummins E. P. (2009). The role of NF-kappaB in hypoxia-induced gene expression. Ann. N. Y. Acad. Sci. 1177, 178–184. 10.1111/j.1749-6632.2009.05024.x 19845620

[B186] Tolle L. B. Standiford T. J. (2013). Danger-associated molecular patterns (DAMPs) in acute lung injury. J. Pathol. 229, 145–156. 10.1002/path.4124 23097158

[B187] Van Hée V. F. Pérez-Escuredo J. Cacace A. Copetti T. Sonveaux P. (2015). Lactate does not activate NF-κB in oxidative tumor cells. Front. Pharmacol. 6, 228. 10.3389/fphar.2015.00228 26528183 PMC4602127

[B188] Vaupel P. Mayer A. Höckel M. (2004). Tumor hypoxia and malignant progression. Methods Enzymol. 381, 335–354. 10.1016/S0076-6879(04)81023-1 15063685

[B189] Végran F. Boidot R. Michiels C. Sonveaux P. Feron O. (2011). Lactate influx through the endothelial cell monocarboxylate transporter MCT1 supports an NF-κB/IL-8 pathway that drives tumor angiogenesis. Cancer Res. 71, 2550–2560. 10.1158/0008-5472.CAN-10-2828 21300765

[B190] Walmsley S. R. Print C. Farahi N. Peyssonnaux C. Johnson R. S. Cramer T. (2005). Hypoxia-induced neutrophil survival is mediated by HIF-1alpha-dependent NF-kappaB activity. J. Exp. Med. 201, 105–115. 10.1084/jem.20040624 15630139 PMC2212759

[B191] Wang D. J. Ratnam N. M. Byrd J. C. Guttridge D. C. (2014). NF-κB functions in tumor initiation by suppressing the surveillance of both innate and adaptive immune cells. Cell. Rep. 9, 90–103. 10.1016/j.celrep.2014.08.049 25263557 PMC4882153

[B192] Wang G. L. Jiang B. H. Rue E. A. Semenza G. L. (1995). Hypoxia-inducible factor 1 is a basic-helix-loop-helix-PAS heterodimer regulated by cellular O2 tension. Proc. Natl. Acad. Sci. U. S. A. 92, 5510–5514. 10.1073/pnas.92.12.5510 7539918 PMC41725

[B193] Wang G. L. Semenza G. L. (1995). Purification and characterization of hypoxia-inducible factor. J. Biol. Chem. 270, 1230–1237. 10.1074/jbc.270.3.1230 7836384

[B194] Warburg Berlin-Dahlem O. (2016). Association for cancer research.

[B195] Warburg O. (1956). Undefined on respiratory impairment in cancer cells. Science 124, 269–270. Available at: https://ci.nii.ac.jp/naid/10030731622/ (Accessed May 17, 2021). 13351639

[B221] Warburg O. (1925). The metabolism of carcinoma cells 1. J. Cancer Res. 9, 148–163. 10.1158/jcr.1925.148

[B196] Wilson D. Rumsey W. Green T. J. Vanderkooi J. M. (1988). Undefined the oxygen dependence of mitochondrial oxidative phosphorylation measured by a new optical method for measuring oxygen concentration. J. Biol. Chem. 263 (6), 2712–2718. Elsevier. Available at: https://www.sciencedirect.com/science/article/pii/S0021925818691264 (Accessed May 17, 2021). 2830260

[B197] Wong C. C. L. Gilkes D. M. Zhang H. Chen J. Wei H. Chaturvedi P. (2011). Hypoxia-inducible factor 1 is a master regulator of breast cancer metastatic niche formation. Proc. Natl. Acad. Sci. U. S. A. 108, 16369–16374. 10.1073/pnas.1113483108 21911388 PMC3182724

[B198] Wu Y. Zhou B. P. (2009). Inflammation: A driving force speeds cancer metastasis. Cell. Cycle 8, 3267–3273. 10.4161/cc.8.20.9699 19770594 PMC3702728

[B199] Xie X. Xiao H. Ding F. Zhong H. Zhu J. Ma N. (2014). Over-expression of prolyl hydroxylase-1 blocks NF-κB-mediated cyclin D1 expression and proliferation in lung carcinoma cells. Cancer Genet. 207, 188–194. 10.1016/j.cancergen.2014.04.008 24935227

[B200] Yang M. H. Wu M. Z. Chiou S. H. Chen P. M. Chang S. Y. Liu C. J. (2008). Direct regulation of TWIST by HIF-1alpha promotes metastasis. Nat. Cell. Biol. 10, 295–305. 10.1038/ncb1691 18297062

[B201] Yoo W. Noh K. H. Ahn J. H. Yu J. H. Seo J. A. Kim S. G. (2014). HIF-1α expression as a protective strategy of HepG2 cells against fatty acid-induced toxicity. J. Cell. Biochem. 115, 1147–1158. 10.1002/jcb.24757 24402912

[B202] You Z. Madrid L. V. Saims D. Sedivy J. Wang C. Y. (2002). c-Myc sensitizes cells to tumor necrosis factor-mediated apoptosis by inhibiting nuclear factor κB transactivation. J. Biol. Chem. 277, 36671–36677. 10.1074/jbc.M203213200 12149248

[B203] Young R. M. Ackerman D. Quinn Z. L. Mancuso A. Gruber M. Liu L. (2013). Dysregulated mTORC1 renders cells critically dependent on desaturated lipids for survival under tumor-like stress. Genes. Dev. 27, 1115–1131. 10.1101/gad.198630.112 23699409 PMC3672646

[B204] Zaidi N. Lupien L. Kuemmerle N. B. Kinlaw W. B. Swinnen J. V. Smans K. (2013). Lipogenesis and lipolysis: The pathways exploited by the cancer cells to acquire fatty acids. Prog. Lipid Res. 52, 585–589. 10.1016/j.plipres.2013.08.005 24001676 PMC4002264

[B205] Zhang H. Bosch-Marce M. Shimoda L. A. Yee S. T. Jin H. B. Wesley J. B. (2008). Mitochondrial autophagy is an HIF-1-dependent adaptive metabolic response to hypoxia. J. Biol. Chem. 283, 10892–10903. 10.1074/jbc.M800102200 18281291 PMC2447655

[B206] Zhang J. Peng B. Chen X. (2005). Expressions of nuclear factor kappaB, inducible nitric oxide synthase, and vascular endothelial growth factor in adenoid cystic carcinoma of salivary glands: Correlations with the angiogenesis and clinical outcome. Clin. Cancer Res. 11, 7334–7343. 10.1158/1078-0432.CCR-05-0241 16243805

[B207] Zhang L. Li L. Liu H. Prabhakaran K. Zhang X. Borowitz J. L. (2007). HIF-1alpha activation by a redox-sensitive pathway mediates cyanide-induced BNIP3 upregulation and mitochondrial-dependent cell death. Free Radic. Biol. Med. 43, 117–127. 10.1016/j.freeradbiomed.2007.04.005 17561100 PMC2048659

[B208] Zhang S. Zhou X. Wang B. Zhang K. Liu S. Yue K. (2014). Loss of VHL expression contributes to epithelial-mesenchymal transition in oral squamous cell carcinoma. Oral Oncol. 50, 809–817. 10.1016/j.oraloncology.2014.06.007 24998140

[B209] Zhi Y. Duan Y. Zhou X. Yin X. Guan G. Zhang H. (2014). NF-κB signaling pathway confers neuroblastoma cells migration and invasion ability via the regulation of CXCR4. Med. Sci. Monit. 20, 2746–2752. 10.12659/MSM.892597 25527973 PMC4280060

