# Supplementary material for: NF-κB mediated regulation of tumor cell proliferation in hypoxic microenvironment
Source: Front Pharmacol. 2023 Feb 20;14:1108915. doi: 10.3389/fphar.2023.1108915 (PMC9986608; doi:10.3389/fphar.2023.1108915)
Supplement: Supplementary file 1 [file Table1.pdf]

**Similarities**		
**Properties**	**IKKβ**	**HIF-1α**
**Basic helix loop helix**	**Present**	**Present**
**Prolyl hydroxylation site**	**Present At P191**	**Present at P402 and P564**
**pVHL binding and proteasomal degradation**	**After prolylhydroxylation at P191 pVHL binds and recruits Ubiquitin by activity of E3 ubiquitin ligase(K48-mediated ubiquitination) which marks IKKB for proteasomal degradation.**	**After prolylhydroxylation at P402 and P564, pVHL binds and recruits Ubiquitin by activity of E3 ubiquitin ligase which marks HIF-1α for proteasomal degradation.**
**LxxLAP motif**	**Consensus sequence found from amino acid 186 to 191 of IKKβ.**<br><br>181 182 183 184 185 186 187 188 189 190 191<br>S F V G T L Q Y L A P	**Consensus sequence found on Both NTAD and CTAD of HIF-1α from amino acid 397 to 402 and from 559 to 564 respectively.**<br><br>NTAD- 392 393 394 395 396 397 398 399 400 401 402<br>K E P D A L T L L A P<br><br>CTAD- 554 555 556 557 558 559 560 561 562 563 564<br>D T D L D L E M L A P

Table 1: Structural Similarities between IKKβ and HIF-1α
